# Protocol for single-cell epigenetic profiling in human organoids and tumoroids with Epi-CyTOF

**DOI:** 10.1016/j.xpro.2026.104695

**Published:** 2026-07-31

**Authors:** Ezgi Senoglu, Vivian Mittné, Sevina Dietz, Anne Eugster, Sebastian Thieme, Franziska Baenke, Mareike Albert, Claudia Peitzsch

**Affiliations:** 1 Center for Regenerative Therapies Dresden (CRTD), TUD Dresden University of Technology, 01307 Dresden, Germany; 2 Department of Visceral, Thoracic and Vascular Surgery, Medical Faculty and University Hospital Carl Gustav Carus, Dresden University of Technology, 01307 Dresden, Germany; 3 National Center for Tumor Diseases (NCT/UCC), 01037 Dresden, a partnership between DKFZ, Faculty of Medicine and University Hospital Carl Gustav Carus, TUD Dresden University of Technology, and Helmholtz-Zentrum Dresden-Rossendorf (HZDR), 01328 Dresden, Germany; 4 German Cancer Consortium (DKTK), Partner Site Dresden, 01307 Dresden, and German Cancer Research Center (DKFZ), 69120 Heidelberg, Germany; 5 Department of Pediatrics, Faculty of Medicine and University Hospital Carl Gustav Carus, Technische Universität Dresden, 01307 Dresden, Germany

**Keywords:** Bioinformatics, cell biology, single cell, mass cytometry, developmental biology, cancer, Genomics, Immunology, molecular biology, Neuroscience, organoids

## Abstract

In this protocol, we present Epi-CyTOF, a cytometry by time of flight (CyTOF) approach for single-cell epigenome profiling in cortical organoids as a model system for human brain development. We describe steps for *in silico* panel design, antibody conjugation and testing, sample preparation and staining, and high-dimensional data analysis. Additionally, we provide recommendations for applying Epi-CyTOF to other model systems based on our experience with gastroesophageal tumoroids.

For complete details on the use and execution of this protocol, please refer to Ditzer et al .^1^

## Highlights

• Protocol for preparing organoid samples for histone modification analysis with CyTOF
• Guidance on antibody panel design, sample barcoding, and cell staining
• Quality metrics for antibody validation and instrument performance
• Standardized workflows for high-dimensional single-cell epigenetic data analysis

## Abstract (editor highlights)

Publisher’s note: Undertaking any experimental protocol requires adherence to local institutional guidelines for laboratory safety and ethics.

## Graphical abstract

**Figure:**
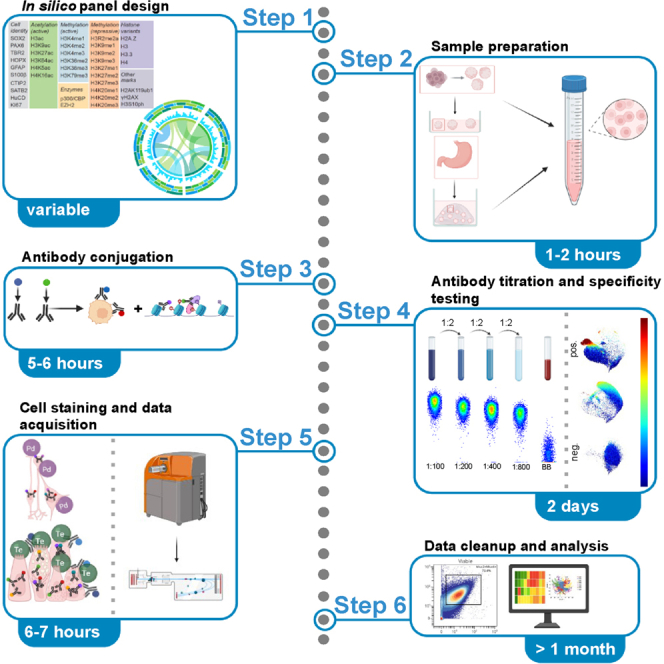

## Before you begin

Within this protocol, cortical organoids and gastro-esophageal tumoroids were used as model systems to explore epigenetic modifications that tightly regulate physiological organ functions and often become dysregulated at the onset of disease. Organoids are 3D model systems derived from induced pluripotent stem cells (iPSCs), embryonic stem cells (ESCs), or directly from adult stem cells of primary tissue.^2^ These models represent important aspects of structural organization, cellular hierarchies, and functional properties for the corresponding tissue. Their applications range from basic developmental biology and regeneration to personalized and translational medicine.

**Figure 1:**
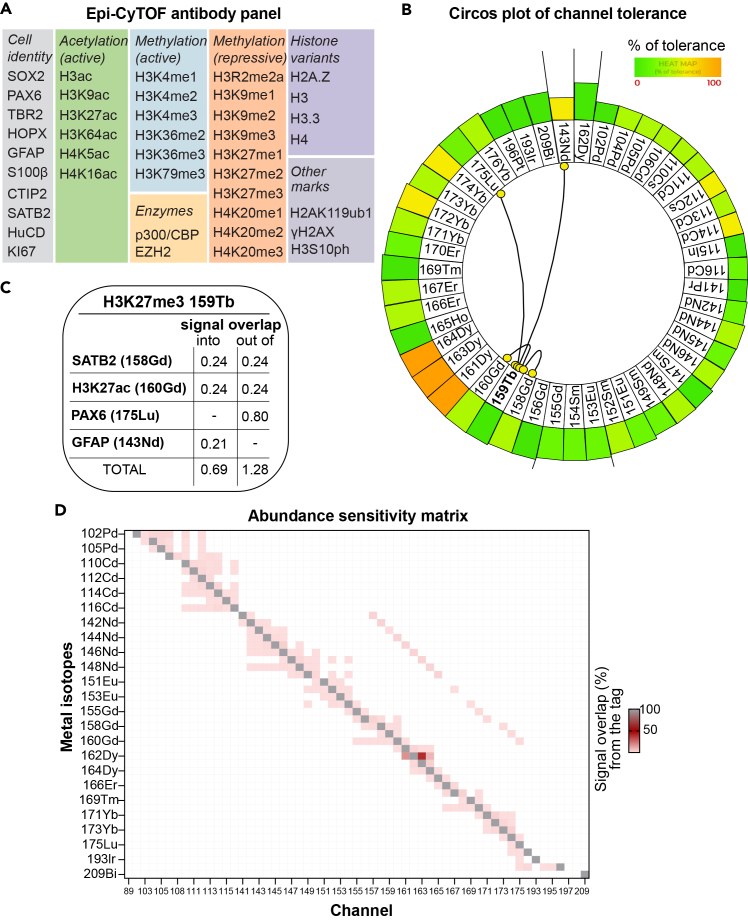
*In silico* panel design and instrument specifications (A) The Epi-CyTOF panel for epigenetic profiling of cortical organoids covers ten cell identity markers, six histone acetylation marks, six activating histone methylation marks, ten repressive histone methylation marks, four histone variants, two epigenetically active enzymes, and three other histone modifications representative of cell proliferation and DNA damage. (B) Overview of the Epi-CyTOF panel as a circos plot using the web-based DVSSciences panel designer. Each metal isotope tag has a specific signal intensity, and each detector has a specific tolerance value. The signal intensity and channel tolerance are known for the palladium (Pd) isotopes available for barcoding and 4 commercially available antibodies. 37 antibodies are custom-conjugated without specifically determined intensity values. Therefore, all self-conjugated antibodies were applied with a signal intensity of 80 and a tolerance value of 16. The illustrative height of each channel tile is proportional to the channel sensitivity. The antibodies are ordered to the ascending tolerance. The color code illustrates putative signal spillover from other channels with different impact (green: low, yellow: median, orange: high). (C) The table shows an example of putative signal contamination into and from the ^159^Tb channel. The spillover is minimal, with a theoretical combined spillover of less than 2%. 1.28% of the total signal may come from the ^158^Gadolinium (^158^Gd), ^160^Gd, and ^175^Lutetium (^175^Lu) channels. High ^159^Tb signals can spill into the ^143^Neodymium (^143^Nd), ^158^Gd, and ^160^Gd channels and account for 0.69% of total signal intensity. Dash indicates that there is no expected spillover. (D) The heatmap illustrates a putative spillover matrix for the Epi-CyTOF panel, depicting the abundance sensitivity of each channel, which is the ratio of the mean metal intensity at M±1 mass channel to the signal at mass channel M. The exact value is instrument-specific and depends on the ion optics. Due to minor effects on channel tolerance and sensitivity, no compensation was applied in this protocol (related to Step 1).

In this study, iPSC-derived cortical organoids were used to investigate the epigenetic regulation of neocortex development.^3^ The neocortex is the brain structure attributed to higher cognitive functions in humans. Its complex and refined structure emerges from a gene expression program tightly regulated by epigenetic mechanisms, including post-translational histone modifications.^4^ These modifications can either activate or repress gene expression, thereby shaping the proliferation and differentiation of neural progenitor cells. Mutations in several genes encoding epigenetic modifiers, such as Trithorax and Polycomb group proteins, disrupt tissue physiology, leading to organ dysfunction and neurodevelopmental disorders, including micro- and macrocephaly.^5^ Animal models have been extensively used to study neocortex development, but there remains a need to employ 3D culture systems to study human-specific features of early developmental processes, particularly in the context of rare human neurodevelopmental disorders. Additionally, given the vast number of histone modifications, our understanding of the epigenetic state of neural cell populations during human brain development remains limited to only a few well-characterized histone modifications.^6^ Within this protocol, we use mass cytometry to investigate global levels of histone modifications and epigenetic enzymes at the single-cell level, in conjunction with neural lineage-specific markers (Figures 1A and 1B).

By utilizing suspension CyTOF, the expression levels of more than 30 different histone modifications and enzymatic writers are measured using a custom-designed antibody panel. This Epi-CyTOF panel explores the levels of epigenetic states at the single-cell level in heterogeneous samples.

To expand its use, we demonstrate how Epi-CyTOF can be applied across various sample types and address different research needs based on the use of gastro-esophageal tumoroids for drug screenings.^7^^,^^8^^,^^9^ There is increasing evidence that epigenetic dysregulation is an important contributor to tumorigenesis in gastrointestinal cancers.^10^ The implementation of epigenetic targeting agents, such as histone deacetylase (HDAC) inhibitors, further enables therapeutic exploitation of epigenetically regulated pathways.^11^

### Innovation

Histone modifications are typically analyzed in bulk, e.g., by Western blotting, mass spectrometry, or chromatin immunoprecipitation (ChIP), resulting in mean values across cells in complex tissues. Recent single-cell epigenomic methods are limited to a few histone modifications.^12^^,^^13^ To achieve single-cell resolution and increase the coverage of epigenetic marks, we apply the previously published CyTOF-based profiling of histone modifications^14^^,^^15^ to human organoid and tumoroid models. To enable broad application of the method, we combined an antibody panel targeting more than 30 epigenetic markers (Epi-CyTOF) with tissue- and lineage-specific markers. The Epi-CyTOF method has several advantages over other epigenetic approaches. Firstly, it combines cellular phenotyping with deep epigenetic profiling, enabling the discovery of high-dimensional epigenetic states in specific cell lineages, including rare populations. Secondly, the rare-metal isotopes used for antibody conjugation remain stable over long periods, enabling sample collection across long-term organoid culture protocols. Thirdly, the preparation and storage of several aliquots prepared from antibody master mixes reduces batch effects. Fourthly, sample multiplexing after cellular barcoding enables high throughput and diminishes technical variations. Fifthly, automated CyTOF measurements and detector voltage optimization on-the-fly enable stable signal detection over long-term measurements. Taken together, Epi-CyTOF represents a powerful multiplexed approach that covzers a large number of epigenetic modifications for investigating complex human tissues or organoid models. Side-by-side comparison of immunohistochemistry and Epi-CyTOF revealed that cell-type-specific changes in histone methylation can be detected by both methods,^1^ yet Epi-CyTOF offers much higher throughput. As such, Epi-CyTOF may guide the selection of epigenetic marks for cell-type-specific epigenomic methods, such as ChIP-based sequencing (ChIPseq) and Cleavage Under Targets and Tzagmentation (CUT&Tag), that resolve the genome-wide distribution of epigenetic modifications.

### Institutional permissions

This study contains human material used to generate cortical organoids and gastro-esophageal tumoroids. All experiments involving human material were performed in accordance with the ethical standards of the institutional and/or national research committee, as well as with the 1964 Helsinki Declaration, and approved by the University Hospital Ethical Review Committee (IRB00001473; IORG0001076; ethical approval number SR-EK-456092021 for cortical organoids, and BO-EK57022020 for gastro-esophageal tumoroids, ovarian tumoroids, and healthy stomach tissue). All participants provided written informed consent before the commencement of the study.

### *In silico* panel design for Epi-CyTOF

**Timing: Variable**

**CRITICAL:** A CyTOF instrument does not have a uniform sensitivity across all channels. The most sensitive mass channel is ^159^terbium (^159^Tb), where low-abundant antigens should be detected. The relative sensitivity drops, e.g., for cadmium or platinum isotopes, which are preferably used to detect targets with high abundance (Figure 1C).

A careful antibody panel design *in silico* is necessary to choose antibody-metal combinations within the mass spectra of the CyTOF between ^89^yttrium (^89^Y) and ^209^bismuth (^209^Bi). Available antibody clones should be selected based on the highest antigen specificity, as determined by previous validation or published literature. When designing a panel, it is crucial to strategically select antibody-metal combinations to achieve maximum signal intensity with minimal background.

***Note:*** Although the signal overlap in mass cytometry is minimal compared to a multicolor flow cytometry panel, an optimized panel design is necessary to obtain high-quality, high-parameter data. For example, the Maxpar Panel Designer (https://pdv2.dvssciences.com/login, software version 2.1.0, user guide #100-9557 Rev 04), a web-based application, can be used. The detector range of the mass cytometer used in this study contains 135 channels between 75 and 209 atomic mass units (amu).

***Note:*** Mass spillover into the neighboring M±1 channel is caused by isotope impurity and abundance sensitivity issues. Although the unspecific signals are less than 0.3% and much lower compared to spectral flow cytometry, they can affect the quantification of marker expression. Another source of unspecific signals is metal oxidation detected within the M+16 channel. Particularly, ^139^lanthanum (^139^La), ^140^cerium (^140^Ce), ^141^praseodymium (^141^Pr), and ^142-144,148^neodymium (Nd) isotopes are sensitive to oxidation with the risk of up to 2% unspecific signal in the mass channel 155 to 164.^16^

**CRITICAL:** To prevent background signals for CyTOF measurements, buffers should not be stored in autoclaved glassware. Detergents used to wash glassware can result in high ^138^barium (^138^Ba) and ^208^lead (^208^Pb) signals. The latter may impede specific ^209^bismuth signal detection. We recommend using commercially available buffers in plastic bottles.

### Preparation of single-cell suspension from cortical organoids

**Timing: 1 h and 10 min**

**CRITICAL:** Live cells are centrifuged at 300 × *g* to preserve cell integrity, and paraformaldehyde (PFA)-fixed cells at 900 x *g*. Centrifugation steps are optimized for the neural cell populations to 7 min.

**Figure 2:**
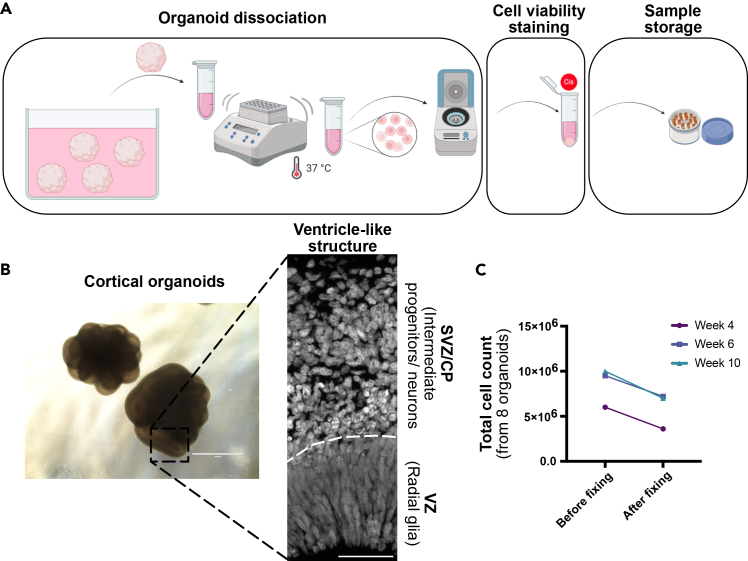
Sample preparation of human cortical organoids for Epi-CyTOF workflow (A) Schematic overview of the separate steps important to prepare the single-cell suspensions of cortical organoids. (B) Brightfield image of six-week-old cortical organoids (left) and immunofluorescence of organoid section depicting tissue architecture (right). Scale bars, 400 μm (left) and 50 μm (right). (C) Line plot showing the cell counts obtained from 8 cortical organoids of various ages, before and after the fixation step during single-cell preparation (related to Steps 1 to 2).

This section will guide you through the preparation of a single-cell suspension from cortical organoid cultures (Figure 2A). The organoids are derived from induced pluripotent stem cells and recapitulate important aspects of human fetal brain development, including cellular diversity, regional organization, and epigenome signatures.^17^ The tissue organization distinguishes the ventricular zone from the subventricular zone, where different neural progenitor cell types reside, and the cortical plate-like region (Figure 2B).

***Note:*** For organoids cultured until week 8, use up to 12 organoids, and for longer cultures maximal 8 organoids per reaction tube. One organoid contains approximately 6 × 10^6^ cells after 4 weeks, 9 × 10^6^ cells at week 6, and 10 × 10^6^ cells at week 10 (Figure 2C).

1. Preparation of single-cell suspensions from cortical organoids with the neural tissue dissociation solution (materials and equipment).
  a. Collect cortical organoids into a 1.5 mL tube and remove any remaining cell media.
  b. Add 1 mL enzyme mix 1 containing 50 μL enzyme P in buffer X to each tube.
  c. Incubate and digest the organoids at 37 °C for 6 min at 300 x *g* in a thermo-shaker.
  d. Add 30 μL enzyme mix 2 containing 10 μL enzyme A in buffer Y and invert the tube to mix.
  e. Dissociate the organoids by pipetting with a P1000 tip.
  f. Centrifuge at 300 × *g* for 5 min to pellet the cell suspension.

**CRITICAL:** Prepare the enzyme mix 1 and 2 of the neural tissue dissociation solution always freshly. All steps are time-critical. Immediately add enzyme mix 2 to tubes containing organoids and proceed with dissociation one by one. If primary human tissue is used instead of organoids, incubate for 15 min. Cell clumps can be removed by filtering the cells through a 40 μm cell strainer (Troubleshooting 1).

***Note:*** Benzonase is added to the cell suspension before staining to digest the free nucleic acid released from dying cells, to avoid cell clumping and reduce cell loss. Cisplatin is added as a cell viability stain, as it covalently binds to DNA and only penetrates cells with a damaged cell membrane. Dead cells would be cisplatin positive and can be removed from analysis.

2. Cell viability staining and preparation of fixed sample stocks.
  a. Resuspend cell pellet in 500 μL cold Dulbecco's phosphate-buffered saline (DPBS).
  b. Add 1 μL Benzonase (25 KU) to prevent cell clumping.
  c. Add 0.7 μL cisplatin (1.25 μM) as a viability dye and incubate for 10 min at 20 to 24 °C. Centrifuge at 300 × *g* for 7 min at 4 °C.
  d. Resuspend each pellet with 2 mL Cell Staining Buffer and centrifuge at 300 × *g* for 7 min at 4 °C.
  e. Resuspend the cell pellet in Maxpar Phosphate Buffered Saline (PBS) containing 1.6% PFA for 10 min at 20 to 24 °C, rotating.
  f. Add 2 mL Cell Staining Buffer and centrifuge at 900 × *g* for 10 min at 20 to 24 °C.
  g. Wash twice with 2 mL Cell Staining Buffer and centrifuge at 900 × *g* for 10 min at 20 to 24 °C.
  h. Count cells and dissolve 1 to 2 × 10^6^ cells in 1 mL freezing media.

**CRITICAL:** The 1.6% PFA solution in PBS needs to be prepared freshly from a 16% stock solution to ensure efficient cell fixation due to repolymerization and oxidation affecting PFA stability. PFA dilutions can be stored at −20 °C up to 6 months. Freeze-thaw cycles should be avoided.

***Note:*** We suggest coating all tubes by washing with a bovine serum albumin (BSA)-containing solution to prevent cells from adhering to the tube wall and reduce cell loss. Counting cells after steps 2 to 5 helps to track cell loss (Figure 2C).

**Pause point:** You can store single-cell suspensions in freezing medium at −80 °C until cell staining.

### Preparation of single-cell suspension from gastroesophageal tumoroids

**Timing: 2 h**

**CRITICAL:** Prepare the tumor dissociation solution, which is used to harvest a single-cell suspension of the gastro-esophageal tumoroids, always freshly. The biobank media that is used to culture the tumoroids can be stored at 4 °C for up to 2 months (materials and equipment).

**Figure 3:**
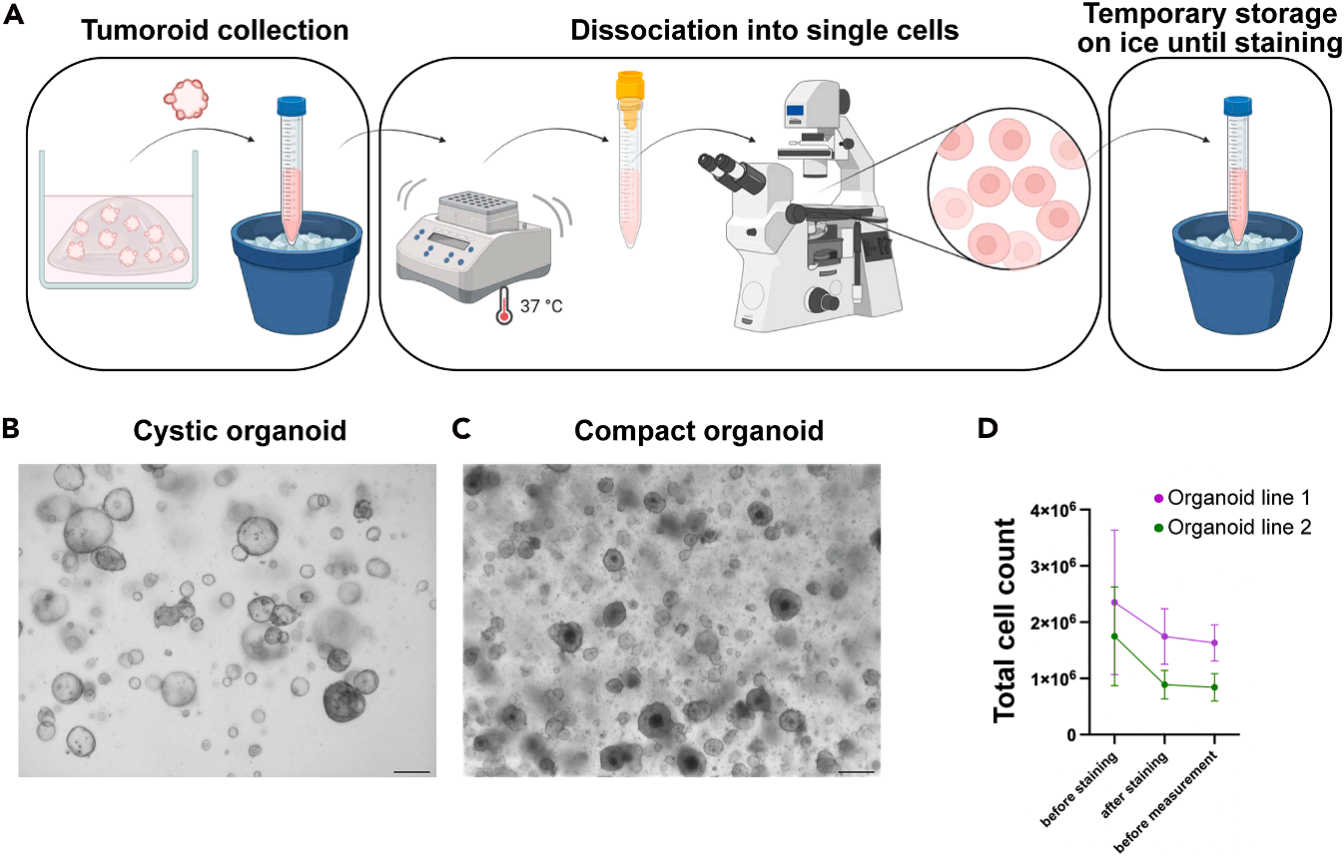
Sample preparation from gastro-esophageal tumoroids for Epi-CyTOF analysis (A) Schematic overview of experimental steps necessary to prepare a single-cell suspension of gastro-esophageal tumoroids. (B) Gastro-esophageal tumoroids exhibit different morphologies representative of tumor histological subtype according to the Laurén classification. Representative brightfield image of a cystic organoid derived from an intestinal-type gastric cancer biopsy (scale bar, 100 μm). (C) Representative brightfield image of a compact organoid with diffuse or mixed histological subtype according to the Laurén classification (scale bar, 100 μm). (D) Cell counts before and after cell staining, as well as after preservation and directly before CyTOF measurements (two tumoroid lines DD485 and DD2058, n = 3, error bar represents standard deviation). A cell loss of up to 60% is observed after permeabilization and intranuclear cell staining. Stained and fixed cells can be preserved at −80 °C without cell loss (related to Step 3).

This section will guide you stepwise through the generation of single-cell suspensions from gastro-esophageal tumoroids (Figure 3A), which were established as previously described.^18^ 3D tumoroids are generated from tumor biopsies or resections of patients with cancers of the gastrointestinal tract, i.e., gastro-esophageal junction, gastric, and colorectal tumors, that are routinely collected during endoscopic examination or surgical resection.^10^ The rate of successful and robust tumoroid growth depends on the tumor cell content within the sample, tissue size, tumor stage, and pre-sampling therapy, and takes between 4 weeks and 3 months.^19^ On average, we obtain a 61% take rate.

***Note:*** For the CyTOF workflow, at least 15 confluent wells of a 48-well plate are used to ensure sufficient cell numbers for analysis. Cell numbers vary between different morphologies of tumoroids (Figures 3B and 3C). On average, 2 × 10^6^ cells are obtained after dissociating organoids from the 15 wells. Depending on the organoid line, between 40 to 60% cell loss was observed during cell preparation and staining (Figure 3D).

3. Preparation of a single-cell suspension of tumoroids.
  a. Harvest tumoroids into a 15 mL tube and wash each well with DPBS.
  b. Centrifuge at 500 × *g* at 4 °C for 5 min.
  c. Resuspend in 1 mL cold Cell Recovery Solution and incubate on ice for 15 min.
  d. Wash with 14 mL cold DPBS and centrifuge to pellet with 500 × *g* at 4 °C for 5 min.
  e. Resuspend the cell pellet with 1 mL TrypLE Express and incubate for a maximum of 40 min at 37 °C with 650 rpm on a thermo-shaker.
  f. Check dissociation status under the microscope every 5 to 10 min (Troubleshooting 2).
  g. Dissociate large clusters by gentle pipetting with a P1000 tip and filter the cell suspension through a 30 μm filter to remove cell debris.
  h. Wash with 14 mL cold DPBS and centrifuge to pellet with 500 × *g* at 4 °C for 5 min.
  i. Resuspend the cell suspension with 500 μL cold DPBS.
  j. Add 1 μL Benzonase (10 KU), 0.25 μL cisplatin (1.25 μM), and 0.5 μL fetal bovine serum (FBS) and incubate for 10 min at 20 to 24 °C.
  k. Wash with 2 mL Cell Staining Buffer and centrifuge at 500 × *g* for 5 min at 4 °C.

**CRITICAL:** Keep the single-cell suspension on ice and process it immediately to prevent cell death and clumping. In contrast to the cortical organoid protocol, cell viability staining and fixation are performed after extracellular staining.

***Note:*** If tumoroids derived from cancer patients treated with a cisplatin-based chemotherapy, the cationic DNA intercalator ^103^rhodium (^103^Rh) should be used as a viability dye.

## Key resources table

**Table:** 

REAGENT or RESOURCE	SOURCE	IDENTIFIER
**Antibodies**		

Rabbit monoclonal anti-EZH2-^144^Nd (clone: D2C9-)	Cell Signaling Technology	Cat: 16098SF; RRID: n.a.
Mouse monoclonal anti-Ki-67-^162^Dy (clone: B56)	Standard BioTools	Cat: 3162012B; RRID: AB_2888928
Rabbit monoclonal anti-H2AK119ub-^153^Eu (clone: D27C4)	Cell Signaling Technology	Cat: 45772SF; RRID: n.a.
Rabbit monoclonal anti-H2A.Z-^148^Nd (Clone: EPR6171)	Abcam	Cat: ab208691; RRID: n.a.
Mouse monoclonal anti-γH2A.X-^174^Yb (clone: JBW301)	Merck Millipore	Cat: 05-636; RRID: AB_309864
Rabbit monoclonal H3-^115^In (clone: D1H2)	IonPath (out of business)	Cat: 711501; RRID: n.a. (discontinued)
Rabbit monoclonal anti-H3-^115^In (clone: D1H2)	Cell Signaling Technology	Cat: 60932SF; RRID: AB_3289584
Rabbit monoclonal anti-H3K4me1-^154^Sm (clone: D1A9)	Cell Signaling Technology	Cat: 5326BF; RRID: AB_10695148
Mouse monoclonal anti-H3K4me2-^173^Yb (clone: MABI 0303)	Active Motif	Cat: 39679; RRID: AB_2793302
Rabbit monoclonal anti-H3K4me3-^145^Nd (clone: C42D8)	Cell Signaling Technology	Cat: 19776SF; RRID: n.a.
Rabbit polyclonal anti-H3ac-^112^Cd	Active Motif	Cat: 39139; RRID: AB_2687871
Rabbit monoclonal anti-H3.3-^113^In (clone: EPR17899)	Abcam	Cat: ab208690; RRID: n.a.
Rat monoclonal anti-H3K9ac-^164^Dy (clone: 2G1F9)	Active Motif	Cat: 61663; RRID: AB_2793725
Mouse monoclonal anti-H3K9me1-^114^Cd (clone: 7E7.H12)	BioLegend	Cat: 824201; RRID: AB_2564878
Mouse monoclonal anti-H3K9me2-^156^Gd (clone: 5E5-G5)	BioLegend	Cat: 815504; RRID: AB_2820126
Rabbit monoclonal anti-H3K9me3-^170^Er (clone: D4W1U)	Cell Signaling Technology	Cat: 13969BF; RRID: AB_2798355
Rabbit monoclonal anti-H3K14ac-^158^Gd (clone: D4B9)	Cell Signaling Technology	Cat: 7627BF; RRID: AB_10839410
Rabbit monoclonal anti-H3K27ac-^160^Gd (clone: D5E4)	Cell Signaling Technology	Cat: 87261SF; RRID: n.a.
Mouse monoclonal anti-H3K27me1-^151^Eu (clone: MABI 0321)	Active Motif	Cat: 61015; RRID: AB_2715573
Mouse monoclonal anti-H3K27me2-^176^Yb (clone: MABI 0324)	Active Motif	Cat: 61435; RRID: AB_2793635
Mouse monoclonal anti-H3K27me3-^159^Tb (clone: MABI 0323)	Active Motif	Cat: 61017; RRID: AB_2614987
Mouse monoclonal anti-H3K27me3-^168^Er (clone: MABI 0323)	Active Motif	Cat: 61017; RRID: AB_2614987
Rabbit monoclonal anti-H3K36me1-^166^Er (clone: EPR16993)	Abcam	Cat: ab208581; RRID: n.a.
Mouse monoclonal anti-H3K36me2-^111^Cd (clone: MABI 0332)	Active Motif	Cat: 61019; RRID: AB_2650523
Rabbit monoclonal anti-H3K36me3-^141^Pr (clone: D5A7)	Cell Signaling Technology	Cat: 4909BF; RRID: AB_1950414
Rabbit monoclonal anti-H3K64ac-^142^Nd (clone: EPR20713)	Abcam	Cat: ab251549; RRID: n.a.
Rabbit monoclonal anti-H3K79me3-^167^Er (clone: EPR17468(2))	Abcam	Cat: ab251493; RRID: n.a.
Rabbit polyclonal anti-H3R2me2a-^163^Dy	Epigentek	Cat: A-3714-100; RRID: AB_2313773
Mouse monoclonal anti-H3S10ph-^171^Yb (clone: MABI 0312)	Active Motif	Cat: 39636; RRID: AB_2793285
Mouse monoclonal anti-H4-^209^Bi (clone: 31830)	Abcam	Cat: ab238663; RRID: n.a.
Mouse monoclonal anti-H4K5ac-^116^Cd (clone: MABI 0405)	Active Motif	Cat: 61523; RRID: AB_2793668
Rabbit monoclonal anti-H4K16ac-^146^Nd (clone: E2B8W)	Cell Signaling Technology	Cat: 37286SF; RRID: n.a.
Mouse monoclonal anti-H4K20me1-^152^Sm (clone: 5E10-D8)	Active Motif	Cat: 39727; RRID: AB_2615074
Mouse monoclonal anti-H4K20me2-^161^Dy (clone: MABI 0422)	Active Motif	Cat: 61533; RRID: AB_2650525
Mouse monoclonal anti-H4K20me3-^149^Sm (clone: 6F8-D9)	BioLegend	Cat: 827701; RRID: AB_2564914
Rabbit monoclonal anti-KAT3B/p300-^165^Ho (clone: EPR23753-55)	Abcam	Cat: ab283721; RRID: n.a.
Rat monoclonal anti-CTIP2-^147^Sm (clone: 25B6)	Abcam	Cat: ab18465; RRID: n.a.
Mouse monoclonal anti-GFAP-^143^Nd (clone: GA5)	Standard BioTools	Cat: 3143022B; RRID: AB_2938640
Rabbit polyclonal anti-HOP-^155^Gd	Novus Biologicals	Cat: NBP2-82093; RRID: AB_3423623
Rabbit monoclonal anti-HuD/C-^166^Er (clone: EPR19098)	Abcam	Cat: ab232416; RRID: n.a.
Mouse monoclonal anti-PAX6-^175^Lu (clone: PAX6/498)	Novus Biologicals	Cat: NBP2-34705; RRID: AB_3291566
Mouse monoclonal anti-S100B-^169^Tm (clone: 15F4NB)	Novus Biologicals	Cat: NBP2-45225; RRID: AB_3309383
Mouse monoclonal anti-SATB1/2-^158^Gd (clone: SATBA4B10)	Abcam	Cat: ab51502; RRID: AB_882455
Mouse monoclonal anti-SOX2-^150^Nd (Clone: O30-678)	Standard BioTools	Cat: 3150019B; RRID: AB_3086680
Rabbit monoclonal anti-TBR2-^172^Yb (clone: EPR21950-241)	Abcam	Cat: ab261913; RRID: AB_2943175
Rabbit monoclonal anti-Cadherin17-^169^Tm (clone: CDH17/8115R)	Novus Biologicals	Cat: NBP3-20885; RRID: AB_3614254
Mouse monoclonal anti-E-Cadherin-^143^Nd (clone: 967738)	R&D Systems	Cat: MAB18383-100; RRID: AB_3657748
Rabbit monoclonal anti-Chromogranin A-^150^Nd (clone: CHGA/1815R)	Novus Biologicals	Cat: NBP2-54363; RRID: AB_3333979
Rabbit monoclonal anti-MUC2-^147^Sm (clone: EPR6144)	Abcam	Cat: ab248551; RRID: n.a.
Mouse monoclonal anti-MUC5AC-^172^Yb (clone: MUC5AC/917+45M1)	Novus Biologicals	Cat: NBP2-47696; RRID: AB_3313966
Rabbit monoclonal anti-MUC6-^159^Tb (clone: EPR20623)	Abcam	Cat: ab251578; RRID: n.a.
Rabbit monoclonal anti-Pepsinogen II/PGC-^155^Gd (clone: EPR22843-77)	Abcam	Cat: ab276148; RRID: n.a.

**Biological samples**		

Human cortical brain organoids	This study	Ditzer et al.^1^
Human ovarian tumoroid	Patient-derived Tumor Model Unit (PMU), National Center for Tumor Diseases Dresden, Germany	DD1745
Human healthy stomach tissue	University Hospital Carl Gustav Carus, Dresden, Germany (Patient-derived Tumor Model Unit (PMU), National Center for Tumor Diseases Dresden, Germany)	DD3068 (DD1972)
Human gastro-esophageal tumoroid	Department of Visceral-, Thoracic- and Vascular Surgery, Medical Faculty Carl Gustav Carus, Dresden, Germany	DD485, DD2058

**Chemicals, peptides, and recombinant proteins**		

A83-01	Tocris Bioscience	2939
Advanced DMEM/F12	Thermo Fisher Scientific	12634010
B27	Invitrogen	17504–044
CryoSure-DMSO	WA-Chemie Medical	WAK-DMSO-10
DMEM/F12	Gibco	31330095
GlutaMAX Supplement	Thermo Fisher Scientific	35050061
HEPES	Thermo Fisher Scientific	15630106
Human [Leu^15^]-Gastrin	Sigma-Aldrich	G9145
Matrigel Phenol-red free	Corning	356231
Mouse recombinant EGF	Invitrogen	PMG8041
N-acetyl-L-cysteine	Sigma-Aldrich	A7250
Nicotinamide	Sigma-Aldrich	72340
Noggin conditioned medium	Provided by Hans Clevers Laboratory	n.a.
Penicillin-Streptomycin	Thermo Fisher Scientific	15140122
Primocin	Invivogen	ant-pm-1
Recombinant FGF-10	Qkine	QKN-QK003
Rspondin 1 conditioned medium	Provided by Hans Clevers Laboratory	n.a.
Wnt3A conditioned medium	Provided by Hans Clevers Laboratory	n.a.
Benzonase	Sigma-Aldrich	E1014
Benzonase	Merck Millipore	70664
Cell Recovery Solution	Corning	354253
Collagenase XI	Sigma-Aldrich	1148090
Dispase II	Roche	04942078001
Dulbecco's Phosphate Buffered Saline (DPBS)	Corning	21-031-CV
Dulbecco's Phosphate Buffered Saline	Sigma-Aldrich	D8537
MACS Neural tissue dissociation kit P	Miltenyi Biotec Inc.	130-092-628
Trypan blue	Thermo Fisher Scientific	T10282
TrypLE™ Express	Thermo Fisher Scientific	12604021
Bovine serum albumin (BSA)	Sigma-Aldrich	A4503
Cell-ID™ 20-plex Pd barcoding kit	Standard BioTools	201060
Cell-ID Cisplatin	Standard BioTools	201064
Cell-ID Intercalator Iridium	Standard BioTools	201192B
Ethylenediaminetetraacetic acid (EDTA)	Sigma-Aldrich	324503
16% Formaldehyde	Thermo Fisher Scientific	28906
Maxpar 10X Barcode Perm Buffer	Standard BioTools	201057
Maxpar Cell Staining Buffer	Standard BioTools	201068
Maxpar Fix I Buffer	Standard BioTools	201065
Maxpar Fix Perm Buffer	Standard BioTools	201067
Maxpar Nuclear Antigen Staining (NS and NP) Buffer Set	Standard BioTools	201063
Maxpar PBS	Standard BioTools	201058
TeMal 7 Isotopes	Standard BioTools	CMF-20100
Dimethyl sulfoxide (DMSO)	Sigma-Aldrich	D2650
Fetal Bovine Serum (FBS)	Sigma-Aldrich	F7524
Antibody stabilizer	CANDOR Bioscience	131050
Bismut(III)-nitrat pentahydrat	Sigma-Aldrich	254150
Bond-breaker TCEP solution	Thermo Scientific	77720
CyTOF2 tuning solution	Standard BioTools	201072
EQ Four-element calibration beads	Standard BioTools	201078
EQ Six-element calibration beads	Standard BioTools	201245
Maxpar washing solution (CyTOF2)	Standard BioTools	201070
Maxpar washing solution	Standard BioTools	201243
Maxpar water	Standard BioTools	201069
Maxpar cell acquisition solution plus	Standard BioTools	201244
Indium (III)chlorid-^113^In	Trace Sciences	n.a.
Indium (III)chlorid-^115^In	Trace Sciences	n.a.

**Critical commercial assays**		

Maxpar MCP9 antibody labeling Kit,^111^Cd∗	Standard BioTools	201111A
Maxpar MCP9 antibody labeling Kit,^112^Cd∗	Standard BioTools	201112A
Maxpar MCP9 antibody labeling kit,^114^Cd∗	Standard BioTools	201114A
Maxpar MCP9 antibody labeling kit,^116^Cd∗	Standard BioTools	201116A
Maxpar X8 multimetal labeling kit^a^	Standard BioTools	201300

**Deposited data**		

Epi-CyTOF-Brain	FlowRepository ID: FR-FCM-Z8DC	http://flowrepository.org/id/FR-FCM-Z8DC
Data analysis workflow	GitHub ID: EPI-CyTOF	https://github.com/Stacheltier/EPI-CyTOF

**Experimental models: Cell lines**		

Human: CRTDi004-A iPSC	Völkner et al.^20^	CRTDi004-A; RRID: CVCL_YR23
Human: HPSI0114i-kolf_2 iPSC	Welcome Trust Sanger Institute, HipSci	WTSIi018-B; RRID: CVCL_AE29
HA-Rspo1-Fc-293T	Hans Clevers Laboratory, Hubrecht Institute	n.a.
Hek293-mNoggin-Fc	Hans Clevers Laboratory, Hubrecht Institute	n.a.
Wnt3a SB/Sylvia Boj, HUB clone5,5 Zeocin	Hans Clevers Laboratory, Hubrecht Institute	n.a.

**Software and algorithms**		

CyTOF Software (v9.0.2)	Standard BioTools	https://standardbio.com/products-services/software/cytof-software-v9-3/
FlowJo (v10.10.0)	BD Bioscience	https://www.flowjo.com/flowjo10/overview
OMIQ	Dotmatics	https://app.omiq.ai/login
GraphPad Prism (v10.6.1)	Dotmatics	https://www.graphpad.com
flowCore package (v2.18.0)	Elliset al^21^	https://bioconductor.org/packages/release/bioc/html/flowCore.html
R: A language and environment for statistical computing (v4.4.2, 2024-10-31)	R Core Team	https://www.r-project.org/
CATALYST (v1.30.29)	Chevrier et al,^22^ Nowicka et al,^23^ Crowell et al^24^	https://bioconductor.org/packages/release/bioc/html/CATALYST.html
SingleCellExperiment (v1.28.1)	Amezquita et al^25^	https://bioconductor.org/packages/release/bioc/html/SingleCellExperiment.html
SummarizedExperiment (v1.36.0)	Morgan et al^26^	https://www.bioconductor.org/packages/release/bioc/html/SummarizedExperiment.html
S4Vectors (v0.44.0)	Pagès et al^27^	https://bioconductor.org/packages/release/bioc/html/S4Vectors.html
Biobase (v2.66.0)	Huber et al^28^	https://www.bioconductor.org/packages/release/bioc/html/Biobase.html
scater (v1.34.1)	McCarthy et al^29^	https://www.bioconductor.org/packages/release/bioc/html/scater.html
dplyr (v1.1.4)	Wickham et al^30^	https://dplyr.tidyverse.org
tidyr (v1.3.1)	tidyr: Tidy Messy Data et al^31^	https://tidyr.tidyverse.org
purrr (v1.1.0)	Wickham et al^32^	https://purrr.tidyverse.org
tibble (v3.3.0)	Müller et al^33^	https://tibble.tidyverse.org
FSA (Fisheries Stock Assessment) (v0.10.0)	Ogle et al^34^	https://fishr-core-team.github.io/FSA/
broom (v1.0.9)	Robinson et al^35^	https://broom.tidymodels.org
ggplot2 (v4.0.1)	Wickham et al^36^	https://ggplot2.tidyverse.org
ggsignif (v0.6.4)	Ahlmann-Eltze et al^37^	https://const-ae.github.io/ggsignif/
BioRender		https://www.biorender.com/
Affinity Photo + Designer (1.10.5.1342)	Serif Ltd.	n.a.

**Other**		

CyTOF2	Standard BioTools (former: DVS Sciences and Fluidigm)	https://standardbio.com/support/instrument-support/helios-and-cytof-2-support/
CyTOF XT	Standard BioTools	https://standardbio.com/products-services/cytof-instrument/
MACS Quant VYB	Miltenyi Biotec	https://www.miltenyibiotec.com/DE-en/
NanoDrop™ 2000	Thermo Fisher Scientific	https://www.thermofisher.com/order/catalog/product/de/de/ND-2000

a The critical commercial assay contains R-buffer, C-buffer, and W-buffer.

**CRITICAL:** The antibody stabilizer, bismuth, yttrium, cisplatin, formaldehyde; the Pd barcoding kit, the Maxpar Fix I Buffer, the Maxpar Fix/Perm Buffer, the Maxpar Nuclear Antigen Staining Buffer, the TeMal 7 Isotopes, the Bond-Breaker TCEP solution, reagents within the Maxpar MCP9 and X8 antibody labeling kit, the Maxpar Washing Solution, and the Benzonase are harmful and toxic substances. Handle all reagents containing these chemicals with protective gloves, and perform the work in a fume hood.

## Materials and equipment

**Table:** Recipe 1. Neural dissociation solution

Reagent	Final concentration	Amount
**Enzyme mix 1 (Neural dissociation kit)**		

Buffer X	n.a	950 μL
Enzyme P	n.a	50 μL
Total	–	**1 mL**

**Enzyme mix 2 (Neural dissociation kit)**		

Buffer Y	n.a	20 μL
Enzyme A	n.a	10 μL
Total	–	**30 μl**

**Table:** Recipe 2. Tumor dissociation solution for gastro-oesophageal tumoroids

Reagent	Final concentration	Amount
Collagenase XI	1 mg/mL	n.a.
Dispase II	0.1 mg/mL	n.a.
DMEM/F12	n.a.	7780 μL
GlutaMAX	1X	100 μL
HEPES	10 mM	100 μL
Primocin	100 μg/mL	20 μL
Total	–	**10 mL**

**Table:** Recipe 3. Biobank medium for gastro-oesophageal tumoroids

**Reagent**	Final concentration	Amount
DMEM/F12	n.a.	48.9 mL
GlutaMAX	1X	500 μL
HEPES	10 mM	500 μL
Primocin	100 μg/mL	100 μL
Total	–	**50 mL**

**Table:** Recipe 4. Extracellular blocking reagent

Reagent	Final concentration	**Amount**
Cell staining buffer	n.a	1 mL
BSA	1% (v/v)	0.01 g
EDTA (0.5 mM)	2 mM	4 μL

**Table:** Recipe 5. Freezing reagent for stained samples

Reagent	Final concentration	**Amount**
DMEM	n.a	7 mL
FBS	20% (v/v)	2 mL
DMSO	10% (v/v)	1 mL

**CRITICAL:** This protocol contains harmful and toxic reagents that have to be handled with care. We recommend protecting yourself by wearing a lab coat and gloves. All steps, including toxic reagents, have to be performed under a chemical hood. Safety data sheets (SDD) outline handling recommendations, e.g., for the antibody stabilizer, bismuth (III) nitrate pentahydrate, yttrium (III), cisplatin, formaldehyde, Maxpar Fix I Buffer, Maxpar Fix Perm Buffer, Maxpar Nuclear Antigen Staining Buffer Set, Tris Carboxy Ethyl Phosphene (TCEP) bond-breaker solution, Maxpar antibody labeling Kits, and Maxpar washing solution.

## Step-by-step method details

### Custom antibody conjugation with metal isotopes

**Timing: 5 h and 30 min**

**CRITICAL:** The preparation of the polymer and antibody (Steps 4 and 5) is performed in parallel. All incubation steps at 37 °C in the heating block are time-sensitive and should not be prolonged. Centrifuge all tubes at maximum speed and room temperature after heating to reduce material loss.

Within this section, we describe the steps for customized conjugation of purified antibodies with different metal isotopes when antibody conjugates are not commercially available. Customized antibody conjugation enables a highly flexible design of CyTOF panels and integration of new antibodies into already established panels.

***Note:*** Commercially available conjugation kits provide metal-chelating polymers that contain functional groups such as maleimide, which react with thiol groups and metal-chelating sites. Pre-loaded polymer is conjugated to the antibody through reactive functional groups, such as cysteine sulfhydryl groups, within the constant region. The antibody conjugation with metal isotopes of the lanthanide series from ^141^praseodymium (^141^Pr) to ^176^ytterbium (^176^Yb) is commercially available (Maxpar® X8 multi-metal labelling kit). The linear (7 mm) X8 polymer (11 kDa) contains the chelator diethylenetriaminepentaacetic acid (DTPA) and is used additionally for the conjugation of isotopes ^89^yttrium, ^113^indium (^113^In), ^115^In, and ^209^bismuth.^38^ The cadmium (Cd) isotopes ^106^Cd, ^110^Cd to ^114^Cd, and ^116^Cd can be conjugated with the Maxpar MCP9 polymer (Standard BioTools, user guide #PRD002 rev 14). The platinum (Pt) isotopes ^194^Pt, ^196^Pt, and ^198^Pt are conjugated without polymer following the X8 labelling protocol.^39^

1. Metal isotope polymer conjugation.
  a. Thaw frozen polymer for 30 min at 20–24 °C and resuspend in 95 μL L-buffer included in the Maxpar conjugation kit.
  b. Add 5 μL metal isotope solution (final concentration: 2.5 mM).
  c. Incubate the polymer-metal mixture for 40 min at 37 °C.
  d. Add polymer mixture on top of a 3 kDa filter tube and centrifuge for 25 min at 12,000 × *g* at 20–24 °C to harvest the metal-polymer.
  e. Wash the polymer with 300 μL C-buffer and centrifuge for 30 min at 12,000 × *g.*
2. Reduction of purified antibody.
  a. Determine the antibody concentration at 280 nm with the NanoDrop.
  b. Check the purity of the antibody solution using the 260/280 nm ratio of the NanoDrop. It should be approximately 0.6.
  c. Add 300 μL R-buffer and 200 μL antibody solution (100 μg) on top of a 50 kDa ultra filter tube.
  d. Centrifuge for 10 min at 12,000 × *g* to wash the antibody.
  e. Prepare 4 mM bond-breaker TCEP in R-buffer.
  f. Add 100 μL TCEP dilution to the purified antibody and incubate for 30 min at 37 °C to reduce the disulfide bonds and expose free thiol groups.
  g. Wash the reduced antibody three times with 400 μL C-buffer and centrifuge for 10 min at 12,000 × *g.*

***Note:*** It is possible to conjugate four to eight antibodies simultaneously. We recommend documenting all lot numbers and concentrations for the antibodies and metal isotopes. On average, 78% of the purified antibodies can be recovered after conjugation.

**CRITICAL:** Aliquot the 10X bond-breaker stock Tris(2-carboxyethyl) phosphine hydrochloride (TCEP, 0.5 M) and store at −20 °C for single use.

3. Combination of the reduced antibody with the metal-loaded polymer.
  a. Resuspend the metal-polymer collected on a 3 kDa filter tube with 60 μL C-buffer (Step 1).
  b. Transfer the polymer to the reduced antibody collected on a 50 kDa filter tube (Step 2).
  c. Incubate for 1 h and 45 min at 37 °C, add 300 μL W-buffer and centrifuge for 4 min at 12,000 × *g.*
  d. Wash three times with 400 μL W-buffer and centrifuge for 10 min at 12,000 × *g.*
  e. Resuspend the metal-conjugated antibody captured on the 50 kDa filter with 80 μL W-buffer.
  f. Place the filter tube inversely into a collection tube and centrifuge for 2 min at 1,000 × *g.*
  g. Measure the concentration of the metal isotope-conjugated antibody using the NanoDrop.
  h. Use the W-buffer as a blank to determine the antibody concentration with the NanoDrop.
  i. Adjust the concentration to 0.5 mg/mL with antibody stabilizer and 0.5% (w/v) sodium azide and store at 4 °C.

***Note:*** Newly conjugated antibodies need to be compared to the previous stock to assess their performance when used for the same study. The long-term stability of custom-conjugated antibodies can be ensured by adding 20% antibody stabilizer. Based on our experience, most custom-conjugated antibodies remain stable for several years. In rare cases, instability and signal loss are detected depending on specific clones and/or antibody-metal combinations (Figure S1**,**troubleshooting 3**)**.

### Antibody titration for nuclear antigens

**Timing: 5 h**

**CRITICAL:** We advise to include 10% additional volume in the calculation of the antibody master mix for cell staining. For the staining of 3 x 10^6^ cells, we calculated, for example, with 110 μL containing 55 μL antibody mixture in Cell Staining Buffer and 55 μL blocking solution to ensure enough volume after filtration. Particularly for titration experiments, the antibody mixture is separately prepared and contains 27.5 μL of backbone antibodies with 3 to 4 lineage-specific markers, as well as 27.5 μL of the 5 to 10 antibodies that need to be titrated.

Within this section, we described the steps for titrating the antibodies to detect the optimal concentration to maximize the separation of the positive signal from the background noise.

***Note:*** All antibodies have to be titrated using a positive control for each marker. It is important to use control samples comparable to the planned study samples to prevent insufficient staining or oversaturation in the experimental setting. An ideal antibody titration experiment should contain backbone antibodies along with the antibodies to be titrated. Backbone antibodies are a set of lineage markers that are added to each tube during titration. This allows the detection of cell type clusters of interest and the detection of true positive signals of titration antibodies in different cell clusters. We suggest including well-known lineage markers with a recommended 1:100 dilution.

***Note:*** All custom-conjugated antibodies are stored in antibody stabilizer solution with a final concentration of 0.5 mg/mL. The concentrations of commercially available antibodies are not given by the companies, and staining is recommended with a 1:100 dilution. Several antibodies can be titrated together. Within this protocol, we titrated extracellular, intracellular, and nuclear antibodies in batches of five to seven.

**Figure 4:**
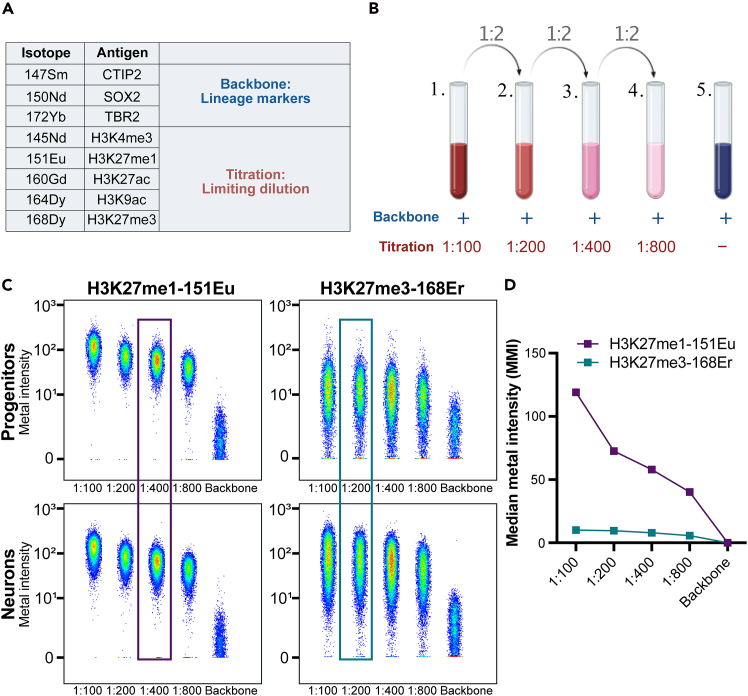
Exemplary antibody titration scheme to determine the optimal concentration of antibodies against histone modifications in cortical organoids (A) For mass cytometry-based applications, five to ten antibodies can be titrated together in one staining reaction. To validate antibody specificity, the antibody titration mixture is combined with three lineage-specific markers as a backbone to separate key cell populations. (B) Schematic overview of dilution series. A cell suspension is split into five aliquots. The 1:100 antibody dilution for titration is prepared for two reactions. One is used for cell staining, and the second for a dilution series. The antibody backbone mixture is added to all five tubes. (C) The analysis software FlowJo (BD Biosciences) is used to separate neural progenitor cells from neurons based on the lineage markers in the antibody backbone mixture. The data from the five dilution steps are concatenated into one file to illustrate the signal intensities of the titrated antibodies across the dilution series. The sample stained only with the backbone lineage markers is negative for the specific antibody staining and determines the background signal within the isotope channel to distinguish negative cells from positive (H3K27me1-^151^Europium (^151^Eu) and H3K27me3-^168^Er). Rectangles indicate the selected antibody concentration for both markers used for Epi-CyTOF staining. (D) The median metal intensity (MMI) of each antibody at different concentrations is illustrated as a titration curve. The limiting dilution series was prepared from a 0.5 mg/mL antibody stock solution and diluted in Nuclear Perm Buffer (related to Steps 4 to 7).
  b. Spin all antibodies at maximal speed for 10 sec to remove precipitations leading to unspecific binding.
  c. Prepare a backbone antibody master mix in Nuclear Perm Buffer for 5 samples (5X of 27.5 μL, total volume: 137.5 μL).
  d. Add 25 μL backbone antibody master mix to the five tubes with the cell suspension and mix.
  e. Add 25 μL Nuclear Perm Buffer to the control tube labelled as backbone.
  f. Prepare the titration antibody master mix in Nuclear Perm Buffer for 2 samples (2X of 27.5 μL, total volume: 55 μL) with 2.2 μL of each antibody that should be titrated (1:100 dilution).
  g. Add 25 μL of the titration antibody master mix to the tube labelled with a 1:100 dilution.
  h. Add 27.5 μL Nuclear Perm Buffer into three tubes for a limiting dilution series.
  i. Prepare a 1:200 dilution of the titration antibody master mix by taking 27.5 μL of the 1:100 dilution and add to the first tube with 27.5 μL Nuclear Perm Buffer.
  j. Continue dilution series two times for a dilution of 1:400 and 1:800 (Figure 4B).
6. Cell fixation and permeabilization for intranuclear staining.
  a. Prepare 1 mL Maxpar Nuclear Staining Buffer by diluting the concentrate 1:4 with diluent solution.
  b. Resuspend the cell pellets with 1 mL Nuclear Staining working solution and incubate for 30 min at 20 to 24 °C to permeabilize cells.
  c. Wash the cells with 2 mL Maxpar Nuclear Perm Buffer and centrifuge at 900 × *g* for 10 min at 20 to 24 °C.
  d. Dilute the cell pellet with 2 mL Maxpar Nuclear Perm Buffer, split into five samples of 400 μL labelled backbone, 1:100, 1:200, 1:400, and 1:800, and centrifuge at 900 × *g* for 10 min at 20 to 24 °C.
  e. Resuspend each cell pellet with 50 μL Nuclear Perm Buffer containing 1% BSA for blocking and incubate 10 min at 20–24 °C.

***Note:*** If samples do not allow harvesting of high cell counts, the cell numbers per titration step can be reduced, but have to be kept equal. If antibodies against low-abundant antigens need to be tested, the cell suspension can be enriched for the population of interest.

4. Viability staining to exclude dead cells from analysis.
  a. Ensure enough cells per titration step to detect rare antigens. If possible, use 1 × 10^6^ cells per titration step. When using a five-step limiting dilution analysis, 5 × 10^6^ cells are needed in total.
  b. Wash cells with 2 mL Cell Staining Buffer and centrifuge at 300 × *g* for 10 min at 4 °C.
  c. Count cells and prepare a cell suspension of 5 × 10^6^ cells in 250 μL Cell Staining Buffer.
  d. Add 0.5 μL live/dead stain ^103^rhodium (5 μM) and incubate for 10 min at 4 °C.
  e. Divide the cell suspension into five tubes with 50 μL (1 × 10^6^ cells) each and label the tubes with the different titration steps.
5. Preparation of backbone and titration antibody master mixes.
  a. Prepare the backbone antibody master mix and select 3 to 4 antibodies against established cell lineage markers with already defined dilutions (Figure 4A).

7. Cell staining with the antibody titration mixtures.
  a. Add 25 μL backbone antibody master mix to all five tubes.
  b. Add 25 μL Nuclear Perm Buffer to the backbone sample to stain only with the lineage markers.
  c. Add 25 μL of the dedicated dilution of the titration antibody master mix (Step 7) to the corresponding samples (1:100, 1:200, 1:400, 1:800) (Figure 4B).
  d. Mix and stain for 1 h on ice.
  e. Wash with 2 mL Cell Staining Buffer and centrifuge at 900 × *g* for 10 min at 4 °C.
  f. Resuspend cell pellets with 1 mL DNA intercalator Iridium (Cell-ID, 125 μM, 1:10,000) in Maxpar Fix Perm Buffer.
  g. Add 125 μL of 16% PFA (final concentration: 4%) and incubate at 4 °C for 14 to 18 h.
  h. Wash with 2 mL Cell Staining Buffer, count the cells of one tube, and centrifuge at 900 × *g* for 10 min at 20 to 24 °C.
  i. Freeze the cell pellets in FBS with 10% dimethyl sulfoxide (DMSO) and store at −80 °C until CyTOF measurements.

***Note:*** The Cell-ID ^191/193^iridium (^191/193^Ir) intercalates within the DNA double-strand, which allows the detection of DNA content in each cell. Compared to flow cytometry, where cells are detected using the FSC/SSC parameters, in CyTOF, the ^191/193^Ir signal is used for event and cell detection, respectively. It is frozen as aliquots of 10 μL and stored long-term at −20 ^o^C. To avoid freezing-thawing cycles, thawed aliquots are kept at 4 ^o^C between staining. Iridium is light-sensitive and needs to be protected. The compound is toxic and irritating and needs to be handled according to the laboratory safety instructions.

**CRITICAL:** The Epi-CyTOF backbone panel can be easily applied to other tissue types when exchanging the lineage markers. However, histone modifications may be differentially regulated across different tissue-specific contexts. Therefore, optimization of the antibody concentration by titrating all antibodies using the same sample type is necessary. Select the antibody concentration according to the best separation of positive and negative populations (Figures 4C and 4D).

### Antibody specificity testing

**Timing: 9 h**

**Figure 5:**
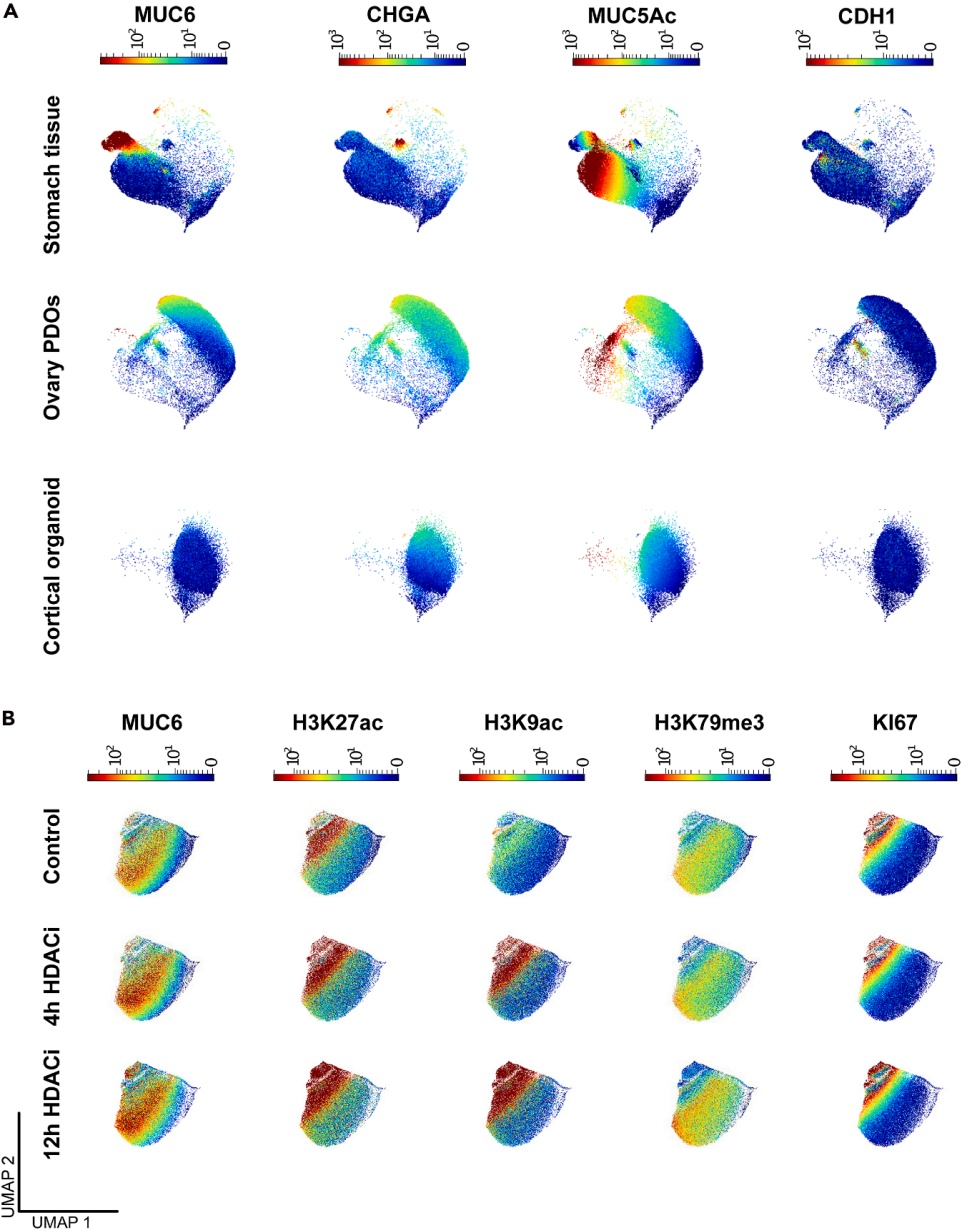
Antibody validation for the Epi-CyTOF staining of gastro-esophageal tumoroids (A) Human healthy stomach tissue, ovarian cancer-derived organoids (PDOs), and cortical organoids are used as positive and negative control samples, respectively, to test the specificity of gastrointestinal lineage markers. Illustrative color-continuous Uniform Manifold Approximation and Projections (UMAPs) show the expression of MUC6, a marker for mucin-producing neck and chief cells in the stomach, chromogranin A (CHGA), an acidic protein expressed in enteroendocrine cells, and MUC5Ac, which is mainly expressed in foveolar cells of the stomach. Cadherin 1 (CDH1, also known as epithelial cadherin, E-cadherin) regulates cell-cell adhesion, cell motility, and proliferation, and is supposed to be expressed by cells in the gastrointestinal tract. (B) To validate antibodies targeting histone acetylation, gastro-esophageal PDOs are treated for 4 and 12 h with the HDAC inhibitor Panobinostat. The color-continuous UMAPs illustrate the increased signal abundance of H3K9 and H3K27 acetylation after HDAC inhibition, whereas histone methylation (H3K79me3), cell proliferation (KI-67), and lineage marker expression (MUC6) are not affected (related to Steps 8 to 10).

**CRITICAL:** The quality of CyTOF data critically depends on the antigen-specificity of the used antibody clones. To exclude non-specific binding, antibody specificity has to be assessed by positive and negative control samples (Figure 5).

In this section, we describe the specificity testing performed for the Epi-CyTOF panel, complementing the extensive validation previously reported by Cheung et al. (2018) and Harpaz et al. (2022).^14^^,^^15^

8. Preparation of a single-cell suspension from healthy stomach tissue as a positive control.
  a. Carefully strip the mucosa from the underlying fatty and muscle tissue (Figure 5A).
  b. Wash the mucosa with DPBS and cut into small fragments using a sterile scalpel.
  c. By using a Pasteur pipette, transfer tissue fragments into a 15 mL tube containing 10 mL tumor dissociation solution (materials and equipment).
  d. Place the tube into a 37 °C water bath and incubate for 15 min to prewarm the digest and soften the tissue.
  e. Incubate for an additional 15 min at 37 °C, shaking at 650 rpm to release stomach glands.
  f. Assess the dissociation status of the stomach glands microscopically.
  g. With a P200 tip, transfer the released glands into a fresh 15 mL tube.
  h. Wash the cell suspension three times with biobank medium and centrifuge at 500 × *g* for 5 min at 4 °C.
  i. Resuspend the visible cell pellet in 1 mL TrypLE Express and incubate at 37 °C with 600 rpm shaking for 30 min.
  j. Filter the single cell solution through a 30 μM cell strainer, wash with DPBS, pellet at 500 x *g* for 5 min at 4°C, and keep the sample on ice until staining.

***Note:*** The preparation of a single-cell suspension from ovarian tumor-derived organoids (PDOs) two days post-splitting follows the procedure for gastro-esophageal tumoroids **(Step 3)**.

9. Extracellular staining of single-cell suspension originating from healthy stomach tissue and ovarian cancer-derived PDOs.
  a. Resuspend each cell suspension with 500 μL cold DPBS, add 1 μL Benzonase (10 KU), 0.25 μL viability dye cisplatin (1.25 μM), and 0.5 μL FBS, and incubate for 10 min at 20 to 24 °C.
  b. Count cells and use 1 to 3 x 10^6^ cells per sample.
  c. Wash with 2 mL Cell Staining Buffer and centrifuge at 300 × *g* for 10 min at 4 °C.
  d. Resuspend cell pellet with 50 μL Cell Staining Buffer containing 1% BSA and incubate for 10 min at RT to block unspecific antibody binding.
  e. Add 50 μL cell surface antibody master mix in Cell Staining Buffer (Table 2), mix, and incubate for 1 h on ice.

**Table 1:** Epi-CyTOF antibody panel for cortical organoids

Antibody	Dilution	Concentration	Cross-reactivity
**Intracellular lineage markers**			

^143^Nd-GFAP (clone: GA5)	1:100	n.a.	H, M, R^b^
^147^Sm-CTIP2 (clone: 25B6)	1:100	10 μg/mL	H, M
^150^Nd-SOX2 (clone: O30-678)	1:100	n.a.	H, M
^155^Gd-HOP (polyclonal)	1:100	10 μg/mL	H
^158^Gd-SATB1/2 (clone: SATB1/2)	1:100	10 μg/mL	H, M
^162^Dy-Ki67 (clone: B56)	1:400	n.a.	H, M, R
^166^Er-HuCD (clone: EPR19098)	1:200	5 μg/mL	H, M, R
^169^Tm-S100 (clone: 15F4NB)	1:100	10 μg/mL	H, M, R
^172^Yb-TBR2 (clone: EPR21950-541)	1:100	10 μg/mL	H, M, R
^175^Yb-PAX6 (clone: PAX6/498)	1:100	10 μg/mL	H, M, R

**Nuclear Epi-CyTOF markers**			

^111^Cd-H3K36me2 (MABI 0332)	1:800^a^	1.25 μg/mL	H
^112^Cd-H3ac (polyclonal)	1:50	20 μg/mL	H
^113^In-H3.3 (EPR17899)	1:50	20 μg/mL	H, M, R
^114^Cd-H3K9me1 (7E7.H12)	1:200	5 μg/mL	H, M
^115^In-H3 (D1H2)	1:50	20 μg/mL	H, M, R
^116^Cd-H4K5ac (MABI 0405)	1:100	10 μg/mL	H
^141^Pr-H3K36me3 (D5A7)	1:50	20 μg/mL	H, M, R
^142^Nd-H3K64ac (EPR20713)	1:400	2.5 μg/mL	H, M, R
^144^Nd-EZH2 (D2C9)	1:100	10 μg/mL	H, M, R
^145^Nd-H3K4me3 (C42D8)	1:800^a^	1.25 μg/mL	H, M, R
^146^Nd-H4K16ac (E2B8W)	1:300	3.33 μg/mL	H, M, R
^148^Nd-H2A.Z (EPR6171)	1:100	10 μg/mL	H, M, R
^149^Sm-H4K20me3 (6F8-D9)	1:200	5 μg/mL	H, M, R
^151^Eu-H3K27me1 (MABI 0321)	1:200	5 μg/mL	H
^152^Sm-H4K20me1 (5E10-D8)	1:100	10 μg/mL	H
^153^Eu-H2Aub119 (D27C4)	1:100	10 μg/mL	H, M, R
^154^Sm-H3K4me1 (D1A9)	1:50	20 μg/mL	H, M, R
^156^Gd-H3K9me2 (5E5-G5)	1:200	5 μg/mL	H, R
^159^Tb-H3K27me3 (MABI 0323)	1:100	10 μg/mL	H, M
^160^Gd-H3K27ac (D5E4)	1:800^a^	1.25 μg/mL	H, M, R
^161^Dy-H4K20me2 (MABI 0422)	1:1600^a^	0.625 μg/mL	H
^163^Dy-H3R2me2a (polyclonal)	1:200	5 μg/mL	H, M, R
^164^Dy-H3K9ac (2G1F9)	1:400	2.5 μg/mL	H
^165^Ho-p300/CBP (EPR23753-55)	1:400	2.5 μg/mL	H, M, R
^167^Er-H3K79me3 (EPR17468(2))	1:100	10 μg/mL	H, M
^170^Er-H3K9me3 (D4W1U)	1:100	10 μg/mL	H, M, R
^171^Yb-phH3 (MABI 0312)	1:200	5 μg/mL	H
^173^Yb-H3K4me2 (MABI 0303)	1:200	5 μg/mL	H, M
^174^Yb-γH2AX (JBW301)	1:1600^a^	0.625 μg/mL	H
^176^Yb-H3K27me2 (MABI 0324)	1:100	10 μg/mL	H
^209^Bi-H4 (31830)	1:100	10 μg/mL	H

a We prepared a pre-dilution of 1:8 in cell staining buffer.

b Abbreviation: H = Human, M = Mouse, R = Rat.

**Table 2:** Epi-CyTOF panel for extracellular markers of gastro-esophageal tumoroids

Antibody	Dilution	Concentration	Cross-reactivity
**Extracellular lineage markers**			

^143^Nd-CDH1 (967738)	1:100	10 μg/mL	Human
^169^Tm-CDH17 (CDH17/8515R)	1:100	10 μg/mL	Human
  f. Wash with 2 mL Cell Staining Buffer and centrifuge at 300 × *g* for 10 min at 4 °C.
  g. Fix with 1 mL 1.6% PFA solution (stock: 16%, diluted in DPBS) for 15 min at 20 to 24 °C.
  h. Wash with 2 mL Cell Staining Buffer and centrifuge at 900 × *g* for 10 min at 4 °C.
10. Cell permeabilization and intranuclear staining.
  a. Prepare a working solution for the Nuclear Antigen Staining Buffer and dilute the concentrate 1:4 with nuclear staining solution.
  b. Resuspend the cell pellets with 1 mL Nuclear Antigen Staining Buffer solution and incubate for 30 min at 20–24 °C for cell permeabilization.
  c. Wash twice with 2 mL Nuclear Perm Buffer and centrifuge at 900 × *g* for 10 min at 20 to 24 °C.
  d. Resuspend the cell pellets with 50 μL Nuclear Perm Buffer, add 50 μL intracellular antibody master mix to each tube (Table 3), and incubate for 1 h at 20 to 24 °C.

**Table 3:** Epi-CyTOF panel for gastro-esophageal tumoroids

ANTIBODY	Dilution	Concentration	CROSS-REACTIVITY
**Intracellular lineage markers**			

^162^Dy-Ki67 (clone: B56)	1:400	2.5 μg/mL	H, M, R^b^
^147^Sm-MUC2 (EPR6144)	1:200	5 μg/mL	H
^150^Nd-CHGA (1815R)	1:100	10 μg/mL	H
^155^Gd-PgC (EPR22843-77)	1:800^a^	1.25 μg/mL	H
^159^Tb-MUC6 (EPR20623)	1:100	10 μg/mL	H
^172^Yb-MUC5AC (Muc5AC/917 + 45M1)	1:100	10 μg/mL	H

**Nuclear Epi-CyTOF markers**			

^111^Cd-H3K36me2 (MABI 0332)	1:800^a^	1.25 μg/mL	H
^112^Cd-H3ac (polyclonal)	1:50	20 μg/mL	H
^113^In-H3.3 (EPR17899)	1:50	20 μg/mL	H, M, R
^114^Cd-H3K9me1 (7E7.H12)	1:200	5 μg/mL	H, M
^115^In-H3 (D1H2)	1:50	20 μg/mL	H, M, R
^116^Cd-H4K5ac (MABI 0405)	1:100	10 μg/mL	H
^141^Pr-H3K36me3 (D5A7)	1:50	20 μg/mL	H, M, R
^142^Nd-H3K64ac (EPR20713)	1:400	2.5 μg/mL	H, M, R
^144^Nd-EZH2 (D2C9)	1:100	10 μg/mL	H, M, R
^145^Nd-H3K4me3 (C42D8)	1:200	5 μg/mL	H, M, R
^146^Nd-H4K16ac (E2B8W)	1:400	2.5 μg/mL	H, M, R
^148^Nd-H2A.Z (EPR6171)	1:100	10 μg/mL	H, M, R
^149^Sm-H4K20me3 (6F8-D9)	1:200	5 μg/mL	H, M, R
^151^Eu-H3K27me1 (MABI 0321)	1:100	10 μg/mL	H
^152^Sm-H4K20me1 (5E10-D8)	1:100	10 μg/mL	H
^153^Eu-H2Aub119 (D27C4)	1:100	10 μg/mL	H, M, R
^154^Sm-H3K4me1 (D1A9)	1:100	10 μg/mL	H, M, R
^156^Gd-H3K9me2 (5E5-G5)	1:200	5 μg/mL	H, R
^158^Gd-H3K14ac (D4B9)	1:200	5 μg/mL	H, M, R
^160^Gd-H3K27ac (D5E4)	1:100	10 μg/mL	H, M, R
^161^Dy-H4K20me2 (MABI 0422)	1:1600^a^	0.625 μg/mL	H
^163^Dy-H3R2me2a (polyclonal)	1:200	5 μg/mL	H, M, R
^164^Dy-H3K9ac (2G1F9)	1:200	5 μg/mL	H
^165^Ho-p300/CBP (EPR23753-55)	1:400	2.5 μg/mL	H, M, R
^166^Er-H3K36me1 (EPR16993)	1:1600^a^	0.625 μg/mL	H, M
^167^Er-H3K79me3 (EPR17468(2))	1:200	5 μg/mL	H, M
^168^Er-H3K27me3 (MABI 0323)	1:200	5 μg/mL	H, M
^170^Er-H3K9me3 (D4W1U)	1:200	5 μg/mL	H, M, R
^171^Yb-phH3 (MABI 0312)	1:200	5 μg/mL	H
^173^Yb-H3K4me2 (MABI 0303)	1:200	5 μg/mL	H, M
^174^Yb-γH2AX (JBW301)	1:1600^a^	0.625 μg/mL	H
^176^Yb-H3K27me2 (MABI 0324)	1:200	5 μg/mL	H
^209^Bi-H4 (31830)	1:100	10 μg/mL	H

a We prepared a pre-dilution of 1:8 in cell staining buffer.

b Abbreviation: H = Human, M = Mouse, R = Rat.
  e. Wash with 2 mL Cell Staining Buffer and centrifuge at 900 x *g* for 10 min at 20 to 24 °C.
  f. Prepare 1 mL Cell-ID Intercalator-Iridium (stock concentration: 500 μM) in Maxpar Fix Perm Buffer (1:10,000 dilution), add 125 μL 16% PFA, and fix at 4 °C for 14 to 18 h.
  g. Wash each sample with 2 mL Cell Staining Buffer and centrifuge at 900 × *g* for 10 min at 20 to 24 °C.
  h. Count cells.
  i. Resuspend the cell pellet in 1 mL FBS containing 10% DMSO, and store at −80 °C until CyTOF measurement.

### Preparation of Epi-CyTOF antibody mixture

**Timing: 2 h**

**CRITICAL:** Calculate approximately 10% additional master mix volume to account for losses due to filtering.

In this section, we describe how to prepare an antibody master mix to be used in an entire study to reduce batch effects.

***Note:*** Before the preparation of the antibody master mix, it is important to determine the total sample number, including the exact cell count per sample, define the barcode pool size, and prepare the required controls, e.g., an anchor sample to spike into each sample pool. Ensure that the same antibody lot number is used throughout the study.

11. Preparation of the antibody master mix and aliquot storage.
  a. Compile an overview of all required antibodies, including their concentration and the volume needed for the entire study.
  b. Calculate the total staining volume based on the number of cells planned for staining.
    i. Prepare the master mix by using Cell Staining Buffer for cell surface antibodies and Nuclear Perm Buffer for intranuclear antibodies.
    ii. Calculate the required volume for each buffer by subtracting the total amount of antibodies from the total staining volume.
  c. Briefly spin all antibody tubes at maximum speed to settle any precipitates, and carefully pipette the solution needed.
  d. Transfer the master mix onto a 50 kDa filter tube and spin at 13,000 x *g* for 2 min to remove precipitates that could affect the staining quality.
  e. Aliquot the master mix according to the planned samples and immediately freeze at −80 °C until use.

**CRITICAL:** The master mixes are stable for several years at −80 °C if recommended storage conditions are followed.^40^ Before using a frozen master mix within a study, we recommend testing the staining performance of freshly prepared versus frozen antibody master mix (Figure S2).

### Palladium-based barcoding of fixed cortical organoids

**Timing: 1 h and 45 min**

**CRITICAL:** The barcode premixes within the Pd barcoding kit are optimized to stain up to 3 x 10^6^ cells. We recommend keeping a consistent cell concentration and, if applicable, equal cell numbers across all pooled samples. Therefore, we prepared aliquots of the commercially available Pd barcodes and reduced the reagent volume according to the available cell count.

Pooling samples before staining minimizes batch effects, reduces technical variation, and decreases antibody consumption. Here we describe the steps for palladium-based barcoding for fixed cortical organoids, which is modified from the manufacturer’s recommended protocol (PRD023 Rev 03), allowing an efficient staining of samples with low cell counts.

***Note:*** Six stable palladium isotopes (^102^Pd, ^104^Pd, ^105^Pd, ^106^Pd, ^108^Pd, and ^110^Pd) are commercially available with purities ranging from 91 to 99%.^41^ The Cell-ID 20-plex Pd barcoding kit uses a 6-choose-3 scheme, allowing multiplexed analysis of up to 20 samples.

12. Cell fixation and permeabilization of cortical organoids.
  a. Thaw the frozen single-cell suspensions (Step 2) and wash each sample with 2 mL Cell Staining Buffer.
  b. Centrifuge the cell suspension at 900 × *g* for 10 min.
  c. Dilute the 5X Fix I Buffer with Maxpar PBS (1:5).
  d. Resuspend the cell pellet in 1 mL Fix I Buffer solution and incubate for 10 min at 20 to 24 °C.
  e. Centrifuge at 900 × *g* for 10 min.
  f. Dilute 10X Barcode Perm Buffer with Maxpar PBS (1:10).
  g. Resuspend the cell pellets in 1 mL Barcode Perm Buffer solution for permeabilization and centrifuge at 900 × *g* for 10 min.
  h. Repeat the permeabilization with 1 mL Barcode Perm Buffer and vortex gently.
  i. Centrifuge the cell suspension at 900 × *g* for 10 min.
13. Preparation of aliquots of palladium barcodes for fixed cells.
  a. Prepare four 2.5 μL aliquots of the 10 μL commercially available barcodes and store immediately at −20 °C until use.
  b. Thaw frozen barcode aliquots quickly and add 25 μL Barcode Perm Buffer to each 2.5 μL barcode.

**CRITICAL:** Frozen barcode mixtures should be thawed rapidly and briefly spun at maximum speed before use.

14. Palladium barcode staining.
  a. Resuspend each cell pellet with 27.5 μL of a defined barcode solution and incubate for 30 min at 20 to 24 °C.
  b. Record all individual barcode schemes along with their respective unique sample ID.
  c. Wash each sample with 1 mL Cell Staining Buffer and centrifuge at 900 × *g* for 10 min.
  d. Resuspend each sample with 100 μL Cell Staining Buffer and combine all samples in one tube.
  e. Add 1 mL Cell Staining Buffer and centrifuge at 900 × *g* for 10 min.
  f. Count cells.
  g. Wash 3 x 10^6^ cells with 2 mL Cell Staining Buffer.
  h. Proceed with a cell concentration of a maximum of 3 x 10^6^ cells per staining reaction of 100 μL for intranuclear staining (Step 22).

### Tellurium-based barcoding of viable gastroesophageal tumoroids

**Timing: 35 min**

***Note:*** We used palladium-based barcoding for fixed samples and tellurium-based barcoding for fresh samples to enable sample pooling for cell staining and measurement. This multiplexing ensures a reduction of batch effects and reagents. We do not barcode the samples when no multiplexing is experimentally planned.

Within this section, we describe the steps for tellurium-based barcoding of viable tumoroid cells using the seven commercially available stable tellurium (Te) isotopes ^122^Te, ^123^Te, ^125^Te, ^126^Te, ^128^Te, and ^130^Te. Tellurophene maleimide (TeMal) penetrates viable cells and covalently binds to free sulfhydryl (thiol) groups in proteins. A 7-choose-3 barcoding scheme enables pooling of up to 35 samples.^42^

15. Preparation of TeMal barcodes.
  a. Prepare TeMal isotope-specific barcodes as a 300 μM stock in Maxpar PBS supplemented with 0.5% DMSO.
  b. Mix the barcodes as a 7-choose-3 scheme containing each 2 μL of three different TeMal isotopes.
  c. Add 4 μL Maxpar PBS to prepare aliquots of 10 μL.
  d. Store at −80 °C until usage.
16. TeMal-based barcoding for viable gastro-esophageal tumoroids.
  a. Resuspend the fresh cell suspension with 300 μL cold Maxpar PBS.
  b. Document the TeMal barcode for each sample ID.
  c. Add 10 μL of a specific TeMal barcode (2 μM) to each sample and incubate for 15 min at 20 to 24 °C.
  d. Wash each sample with 2 mL Cell Staining Buffer and spin at 300 x *g* for 10 min at 4 °C.
  e. Resuspend cell pellets with 1 mL Cell Staining Buffer and pool all samples in one tube.
  f. Count the cell pool and centrifuge at 300 × *g* for 8 min at 4 °C.
  g. Proceed with cell surface staining (Step 23).

### Epi-CyTOF staining of human cortical organoids

This section will guide you through the process of nuclear staining of cortical organoid single cell suspensions.

**Timing: 3 h and 15 min**

17. Thawing of preserved cell suspensions of cortical organoids.
  a. Quickly thaw cryovials with fixed cell suspensions of cortical organoids (Step 2) in a water bath at 37 °C.
  b. Transfer the cells into 15 mL tubes and wash with 2 mL Cell Staining Buffer.
  c. Count cells.
  d. Centrifuge cell suspension at 900 × *g* for 10 min at 20 to 24 °C.
18. Permeabilization of fixed cortical organoid suspension.
  a. Prepare Maxpar Nuclear Antigen Staining Buffer by adding one part of the 4X concentrate to three parts of the dilution solution.
  b. Resuspend the cell pellet with 1 mL Nuclear Antigen Staining Buffer and incubate for 30 min at 20 to 24 °C.
  c. Add 2 mL Nuclear Antigen Perm Buffer and centrifuge at 900 × *g* for 10 min.
  d. Resuspend cell pellet with 2 mL Nuclear Antigen Perm Buffer and centrifuge at 900 × *g* for 10 min.

***Note:*** Cell permeabilization for nuclear staining can be done with different reagents such as methanol, saponin, or the commercially available Nuclear Antigen Perm Buffer. However, the staining quality has to be confirmed by a test sample with known antigen expression (Figure S3, Troubleshooting 4).

19. Nuclear Epi-CyTOF cell staining.
  a. Prepare Nuclear Antigen Perm Buffer with 1% BSA to block unspecific antibody binding.
  b. Dissolve cell pellet with 50 μL Nuclear Antigen Perm Buffer and incubate at 20 to 24 °C for 10 min.
  c. Thaw the frozen, pre-mixed antibody cocktail at 20 to 24 °C (Step 13, Table 1).
  d. Add 50 μL antibody cocktail to the cell suspension and incubate at 20-24 °C for 1 h while mixing regularly.
  e. Add 2 mL Cell Staining Buffer to wash the cell suspension and centrifuge at 900 × *g* for 10 min at 20 to 24 °C.
  f. Prepare 1 mL Cell-ID Intercalator-Ir in Fix Perm Buffer by adding 1 μL iridium (125 μM) to 1 mL buffer (1:2000, final concentration: 62.5 nM).
  g. Resuspend the cell pellet with Cell-ID solution, add 125 μL 16% PFA, and fix cells at 4 ^o^C for 14 to 18 h.
  h. Wash the suspension with 2 mL Cell Staining Buffer and centrifuge at 900 × *g* for 10 min.
  i. Before proceeding with CyTOF measurement, wash the cells with 2 mL MaxPar water.
  j. Resuspend the stained cells in 1 mL freezing reagent containing FBS, 10% DMSO, and store immediately at −80 °C (materials and equipment).

**Pause point:** You can store stained single-cell suspensions in freezing reagent at −80 °C until CyTOF measurements.

***Note:*** All custom-conjugated antibodies are prepared with a stock concentration of 0.5 mg/mL. The concentration of the commercially available antibodies is not known.

### Epi-CyTOF staining of human gastroesophageal tumoroids

**Timing: 4 h**

***Note:*** We tested the Epi-CyTOF antibody concentrations determined with cortical organoids on human gastro-esophageal tumoroids. Most of the antibodies could be used with the same concentration as optimized for the cortical organoids. However, some antigens were present less or so strongly that the antibody concentration was adjusted (Table 3). Therefore, we highly recommend testing the given antibody concentrations beforehand when analyzing another sample source.

In this section, we guide you through the adaptation of the Epi-CyTOF panel to analyze epigenetic states of gastro-esophageal tumoroids and describe the steps for the staining. For that, the neural lineage markers were replaced by markers for epithelial cells with the cadherins CDH1 and CDH17, goblet cells with MUC2, enteroendocrine cells with chromogranin A, pepsinogen II expressing chief cells with progastricsin (pepsinogen C, PGC), and mucous cells with mucin 5AC and mucin 6.

20. Extracellular staining of gastro-esophageal organoid cell suspensions.
  a. Wash single cell pellet (Step 3) with 2 mL Cell Staining Buffer and centrifuge at 500 × *g* for 5 min.
  b. Resuspend 3 x 10^6^ cells with 50 μL Extracellular Blocking Reagent containing Cell Staining Buffer, 1% BSA, and 2 mM EDTA (stock solution: 0.5 M, pH 8) (materials and equipment).
  c. Incubate for 10 min at 20–24 °C to block unspecific antibody binding.
  d. Add 50 μL extracellular antibody mix (Table 2) and mix gently.
  e. Stain cells for 1 h on ice and mix regularly to prevent cells from settling down.
  f. Wash twice with 2 mL Cell Staining Buffer and centrifuge at 300 × *g* and 4 °C for 8 min.
21. Cell fixation and nuclear permeabilization.
  a. Loose cell pellet and resuspend with 1 mL freshly prepared 1.6% methanol-free PFA diluted in Maxpar PBS (1:10 from the 16% PFA stock).
  b. Fix cells for 15 min at 4 °C.
  c. Wash cell suspension twice with 2 mL Cell Staining Buffer and centrifuge at 900 × *g* for 8 min.
  d. Dilute the 4X Nuclear Antigen Staining concentrate with Nuclear Antigen Staining dilution (1:4) to prepare the Nuclear Antigen Staining Buffer.
  e. Resuspend the pellet with 1 mL Nuclear Antigen Staining Buffer and incubate at RT for 30 min.
  f. Wash twice with 2 mL Nuclear Antigen Perm Buffer and centrifuge at 900 × *g* for 10 min at 4 °C.

**CRITICAL:** Sample fixation with PFA may alter the conformation of extracellularly expressed antigens, leading to reduced detection or unspecific higher signals. Therefore, we recommend comparing the staining of extracellular markers after PFA fixation with freshly stained cell suspension (Figure S4, troubleshooting 5).

22. Intranuclear Epi-CyTOF staining of gastro-esophageal tumoroids.
  a. Resuspend the cell pellet in 50 μL Nuclear Antigen Perm Buffer.
  b. Add 50 μL frozen intracellular antibody master mix (Step 14) and incubate for 1 h at 4 °C.
  c. Wash cells with 2 mL Cell Staining Buffer and centrifuge at 900 × *g* for 8 min.
  d. Fix and stain the cell suspension with 1 mL Cell-ID Intercalator-Iridium solution (500 μM stock, 1:10,000) in Fix Perm Buffer.
  e. Add 125 μL 16% PFA and fix cells at 4 ^o^C for 14 to 18 h.
  f. Wash with 2 mL Cell Staining Buffer and centrifuge at 900 × *g* for 8 min.
  g. Cryopreserve the stained cells in 1 mL FBS containing 10% DMSO at −80 ^o^C.

**CRITICAL:** The Cell-ID™ Intercalator-Iridium is commercially available as 125 μM and 500 μM stock. Depending on the stock concentration and sample, the iridium needs to be diluted differently to ensure a staining in Fix Perm Buffer with a final concentration of 62.5 nM. For samples with increased DNA content, i.e., tumor samples, iridium titration is recommended to prevent signal interference affecting surrounding metal isotope channels and detection failure. We normally adjust the concentration to obtain signals between 10^2^ and 10^3^ medium metal intensity (MMI). For the gastro-esophageal tumoroids, we use a reduced concentration of 50 nM for the Cell-ID staining. In comparison, cortical organoids were stained with 62.5 nM.

### Daily quality control before CyTOF measurements

**Timing: 1 h**

***Note:*** Putative sources of background signals may originate from soap-washed plastic bottles, glassware, latex gloves, calcium (Ca^2+^) and magnesium (Mg^2+^) containing PBS, the density gradient solution Ficoll, impure water, and the argon gas tank. Contaminated samples will exhibit a high signal for argon dimers (^40^Ar^40^Ar, m/z 80), ^120^tin (^120^Sn), ^127^iodine (^127^I), ^131^xenon (^131^Xe), ^134/138^barium (^134/138^Ba), and ^208^lead (^208^Pb), and the isotope channels should be monitored for all samples.

Within this section, we describe how to perform daily quality control of CyTOF instruments before samples measurement. This includes instrument start-up, cleaning, background check, and sensitivity testing.

**Figure 6:**
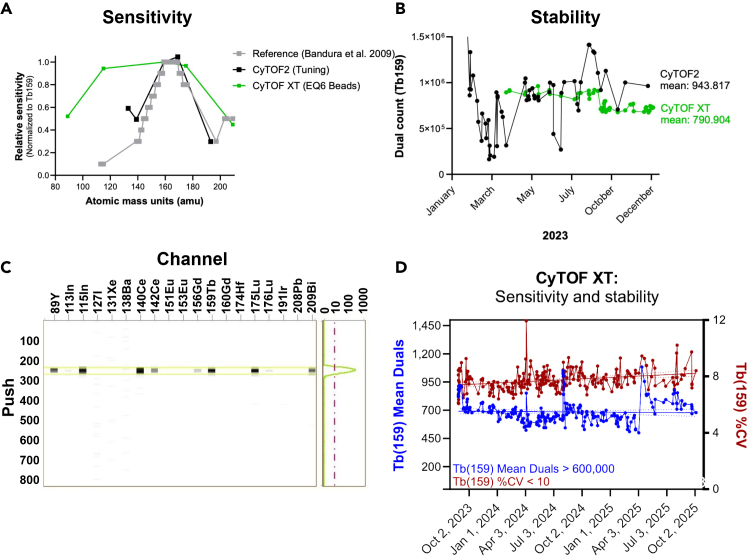
Daily quality control to ensure stable measurements for the CyTOF XT (A) CyTOF measurements can be performed on two machines, namely CyTOF2 or CyTOF XT. Compared to the CyTOF2, the sensitivity of the CyTOF XT at the outer edge of the panel is increased. (B) Based on the daily quality control and tuning results, the CyTOF XT enables more stable measurements. The instrument stability is represented by the dual counts of ^159^Tb, the most sensitive channel. (C) Rain plot to illustrate six-element calibration (EQ6) bead signal for the metal isotopes ^89^Yttrium, ^115^Indium, ^140^Cerium, ^159^Terbium, ^175^Lutetium, and ^209^Bismuth for CyTOF XT tuning. (D) CyTOF XT instrument calibration values and stability over the period of the Epi-CyTOF study, determined by the leading mass channel ^159^Terbium (two years from 2023 to 2025, simple linear regression, 95% confidence interval). Mean dual counts represent the actual ion counts and signal intensity measured by the detector per 13 μsec push after time-of-flight separation. Coefficient of variation (CV) resembles the variance and reproducibility of signal detection (related to Steps 23 to 25).

**CRITICAL:** Six-element calibration beads (EQ6) with a known concentration of ^89^yttrium (^89^Y), ^115^indium (^115^In), ^140^xerium (^140^Ce), ^159^terbium (^159^Tb), ^175^lutetium (^175^Lu), and ^209^bismuth (^209^Bi) are used to optimize the isotope detection, the argon gas flow, rate, and measure putative isotope oxidation (Figures 6A and 6C). The obtained linear list mode data (LMD) files contain the information about the flow rate, the background file (BG.fcs) illustrates putative instrument contamination, and the txt log files store the information about the instrument pressure.

23. Preparation and warm-up of mass cytometer CyTOF XT.
  a. Check argon gas pressure, insert the bottle with Maxpar Cell Acquisition solution plus (CASplus), and start CyTOF software.
  b. Connect the nebulizer and check the spray that fine droplets are formed.
  c. Run a heavy wash, start the plasma, and wait for 20 min.

**CRITICAL:** The EQ6 beads have to be stored at 4 ^**o**^C and have to be vortexed rigorously before usage.

24. Automated tuning and sensitivity testing.
  a. Run the background check with 1 mL CASplus solution. Save as a.fcs file and take a screenshot of the raindrop plot for documentation.
  b. Add 2 mL EQ six-element calibration beads and start the automated tuning program.
  c. Document the results for the detector voltage, the oxide ratio, the mean dual counts, including the CV for ^159^Tb, and the current (Figures 6B and 6D).
  d. Track the values over time to illustrate instrument stability.

***Note:*** The tuning passes when the dual count of ^159^terbium (^159^Tb) is higher than 500.000 with a maximal bead coefficient of variation (CV or relative standard deviation, RSD) of less than 15%, and an oxidation ratio below 3%. The oxidation value is determined by the ^155^gadolinium (^155^Gd) signal after ^139^lanthanum (^139^La) oxidation.

**CRITICAL:** If the daily quality control fails, a stepwise troubleshooting procedure must be performed according to the instrument guidelines and documented in detail (Troubleshooting 6). We routinely check the nebulizer assembly, the connecting lines, and the spray. If the oxidation rate is too high, we check the makeup gas pressure and the argon connection lines.

25. Daily CyTOF instrument cleaning.
  a. Run the long clean program between samples, and an extensive cleaning program before plasma shut-down.
  b. Remove the nebulizer and incubate for 14 to 18 h in 10% Contrad 100.
  c. Rinse the nebulizer with ultrapure water and leave it in water until the next measurements.
  d. Clean the sample injection tubes every week with BD FACS Clean solution and rinse afterwards with water.
  e. Remove injector once per week and clean for 14 to 18 h with 10% Contrad 100-based cleaning solution. Rinse with water afterwards.
  f. Remove and clean cones when detector voltage increases.

**CRITICAL:** If the current is 10, the cones have to be cleaned.

### Data acquisition with CyTOF

**Timing: Variable (approximately 1–2 h per 1 × 10**^**6**^**cells)**

**CRITICAL:** Dissociated epithelial tissue samples or organoids, which tend to form clogs, were measured with a reduced concentration of 0.3 × 10^6^ cells/mL.

This section includes the necessary steps for data acquisition with the mass cytometers CyTOF2 and CyTOF XT. In this study, the CyTOF XT was primarily used.

***Note:*** For the automated measurements with the CyTOF XT, the samples were placed as cell pellets in a cooled carousel and automated resuspended with CASplus solution to a defined concentration of 0.6 x 10^6^ cells/mL. After the addition of 10% EQ6 beads, 250 μL aliquots per cell suspension are injected into the sample loop and measured with a constant event rate of 350 to 500 events/sec.

26. Prepare stained and preserved samples for CyTOF measurements.
  a. Thaw preserved samples in a water bath at 37 °C (Steps 21 and 24).
  b. Wash cells with 2 mL Cell Staining Buffer and centrifuge at 900 × *g* for 10 min.
  c. Resuspend the cell pellet with 2 mL Maxpar water and filter cells through a cell strainer with a 30 μm pore size.
  d. Count the cells with a MACSQuant (Miltenyi Biotec).
    i. Use the total event count to calculate the defined concentration of 0.6 x 10^6^ cells/mL.
    ii. Export the data as FCS and PDF file.
    iii. Inspect the forward (FSC) and side scatter (SSC) gate to document sample quality, e.g., the percentage of dead cells, debris, or doublets.
  e. Prepare aliquots of 2.4 x 10^6^ cells per 5 mL round-bottom, polypropylene tube and centrifuge at 900 × *g* for 10 min.

**CRITICAL:** All suspension samples have to be filtered before measurement to ensure stable measurements. Low-quality samples, e.g., with debris or cell clumps, tend to clog the nebulizer and sample line.

**Figure 7:**
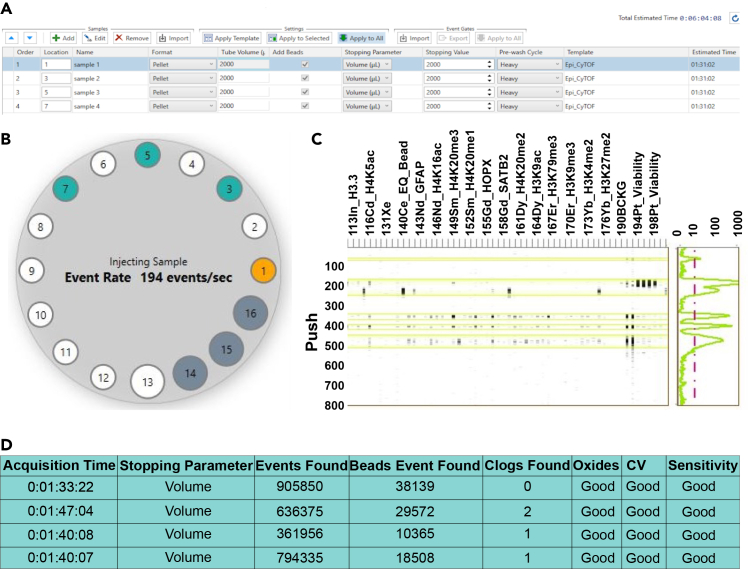
Screenshot of the CyTOF software during sample acquisition (A) Acquisition setup to define sample ID, volume of CASplus solution to ensure a measurement with a constant cell concentration of 3 x 10^6^ cells/mL for the dissociated organoid samples, the addition of 10% EQ6 beads as internal standard, and the defined panel template to ensure acquired data for all necessary metal isotope channels. (B) Automated measurements are enabled through a cooled carousel where samples are placed as pellets. Samples are measured with a constant flow rate of 30 μL/sec in 250 μL aliquots and with an event rate between 150 and 300 events/sec. Sample positions 1 to 12 can be used for 5 mL tubes. Position 14 is for bigger sample volumes of up to 15 mL. In positions 15 and 16, tubes for EQ6 and EQ4 beads are placed, respectively. In this scheme, positions 1, 3, 5, and 7 are filled with samples, and position 1 is selected (marked as orange) according to the acquisition template shown in A. (C) Rain plot during Epi-CyTOF measurements for cortical organoids. The x-axis shows all metal isotope channels, and the y-axis shows the 13 μs pushes. Dual count signals from each channel per push are shown as black raindrop-like signals. Signals above the threshold (dashed red line) with an event duration of 10 to 100 pushes are recorded as an event (green histogram). (D) The generated run report documents the detected events and EQ6 beads, the detected and automatically removed clogs, the oxide ratio, the CV, and the sensitivity (related to Steps 26 to 28).
  c. Check the antibody panel carefully that all markers, EQ beads, and contamination channel are activated and correctly named.
  d. Save the panel template and do not change it throughout the study.
  e. Calculate the necessary volume of EQ6 beads for the run, shake vigorously, and add to the 10 mL tube in the carousel.
  f. Place all samples as a pellet in the correct carousel position according to the information in the acquisition template (Figure 7B).
  g. Start measurements and save screenshots of the CyTOF software manually during the run to document the metal isotope signals in the rain blots, the event rate, and instrument pressure.
  h. Run long cleaning after measurements and automated plasma shut-down at the end.

***Note:*** Ensure sufficient free disk space on the hard drive. Approximately 20 GB of storage is needed per hour of measurements.

27. Measure samples with the suspension mass cytometer CyTOF XT.
  a. Prepare a sample acquisition template within the CyTOF software.
  b. In the acquisition template, define the parameters to estimate the total time (Figure 7A).
    i. Enter the sample name.
    ii. Choose the cell pellet as the sample format.
    iii. Define the sample volume and activate the button to add 10% EQ6 beads automatically.
    iv. Activate a heavy wash between sample aliquots.
    v. Add the project-specific panel template.

**CRITICAL:** The CyTOF software automatically monitors the pressure of the fluidic system for clog detection and initiates a clog removal program in case of clog detection. We typically allow a maximum of two clog removal cycles before replacing the nebulizer (Troubleshooting 7).

28. Data acquisition and conversion.
  a. The rain blot shows the dual count signals within each isotope channel per ion cloud (Figure 7C).
  b. The raw mass spectrometry data are saved as list mode data (LMD) files containing raw ion count data for all isotope channels per 13 μs push reading.
  c. The raw time-of-flight (TOF) data containing the ion spectrum per reading is saved as.txt file format.
  d. List Mode Data (LMD) files are automatically converted to flow cytometry standard (FCS) files containing all metal isotope intensities for every event. Both raw data types can recover the FCS files for data analysis and are routinely saved on tapes for long-term storage.
  e. The raw FCS files are automatically normalized according to the EQ6 bead signals by the CyTOF software.
  f. Use the obtained processed FCS files for downstream de-barcoding, clean-up, and analysis.

***Note:*** An ion cloud consists of several 13 μs pushes, which are separately measured in the TOF chamber. A single cell event is defined by metal isotopes signals above the threshold of 10 and with a duration of 10 to 150 pushes.

### De-barcoding of pooled samples after measurement using the CyTOF software

**Timing: 15 min**

***Note:*** Use the processed FCS file of the pooled sample to separate the individual FCS files for each sample automatically based on the defined barcode. The de-barcode function in the CyTOF software uses two parameters, barcode separation and the Mahalanobis distance, to match barcodes to their respective sample IDs.

This section guides you through the steps to de-barcode the samples that are previously barcoded and pooled, to stain and measure one pool sample to reduce technical variation and batch effects.

**Figure 8:**
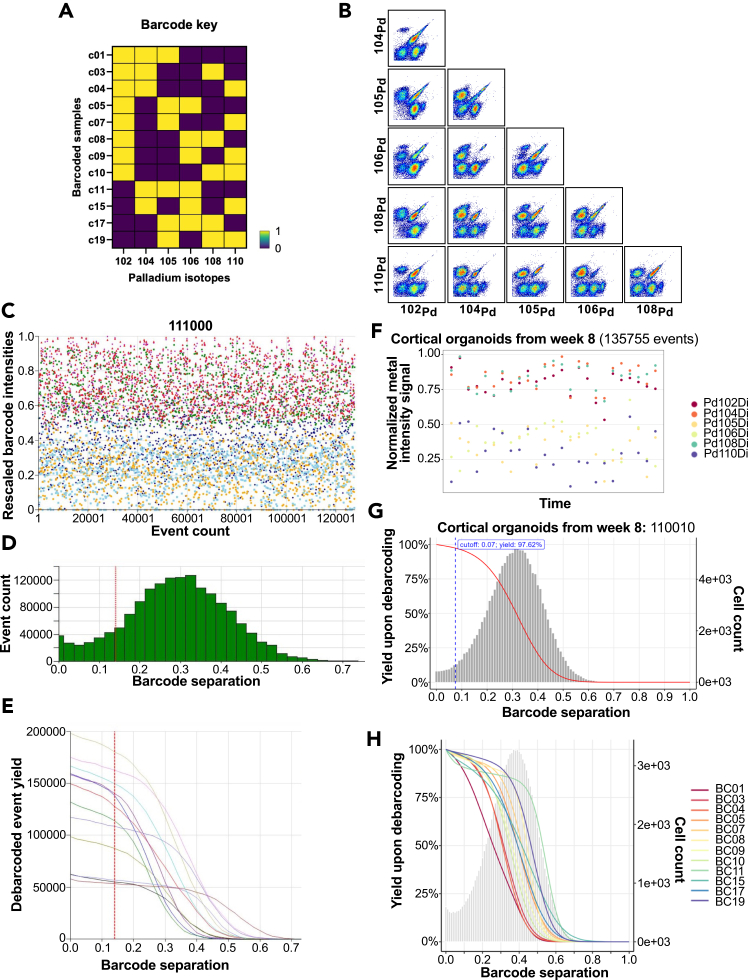
De-barcoding of palladium-barcoded cortical organoids (A) 6-choose-3 barcode scheme for 12 cortical organoid samples. (B) Illustrative histogram and dot blot to demonstrate barcode staining and separation of the 12-plex pool sample. C to F) De-barcoding with the CyTOF software that is based on the Premessa code. (C) Barcode separation for the samples encoded with the code c01 (11100) positive for the isotopes ^102^Pd, ^104^Pd, and ^105^Pd (red, pink, green), and negative for ^106^Pd, ^108^Pd, and ^110^Pd (yellow/purple/light blue). (D) The barcode separation was set to a threshold of 0.14 for all 12 samples. (E) The de-barcoding efficacy and event yield differ between samples due to differing event counts and intensities. (F and H) R-based de-barcoding with the CATALYST package for barcode separation with individually defined thresholds per sample. (F) Barcode separation for the sample encoded with the barcode c01 (110010) positive for the isotopes ^102^Pd, ^104^Pd, and ^108^Pd (red, orange, green), and negative for ^105^Pd, ^106^Pd, and ^110^Pd (yellow, purple, light orange). (G) The barcode cutoff was set automatically to 0.07 with a yield of 97.61% of the acquired events. (H) Summarizing histogram of all samples with individually defined de-barcoding thresholds. Based on the individually set cutoffs per sample, the event yield is higher with this methodology. Moreover, debris and doublets are removed sample-wise (related to Steps 29 to 30).

***Note:*** The minimum barcode separation measures the intensity differences among the three positive and three negative palladium isotopes in the 6-choose-3 scheme (Figures 8A and 8B). This produces a Gaussian distribution, and the threshold is manually set to exclude samples with low separation, such as debris or aggregates (Figure 8C). The maximum Mahalanobis distance represents the data points from the center of the distribution to identify outliers and was set at a value of 10.

29. De-barcoding using the CyTOF software (version 9.2).
  a. Concatenate the processed FCS files obtained per sample aliquot to generate one FCS file for the pooled sample.
  b. Prepare a comma-separated value (CSV) file including the barcode key and the respective sample ID.
  c. Open the de-barcode function in the CyTOF software, assign the barcode key with the barcode manager, and open the concatenated FCS file of the pooled sample.
  d. Define the minimum barcode separation and save the individual FCS files per sample.

***Note:*** If the de-barcoding efficacy is low, the documented barcode ID for each sample has to be double-checked to exclude barcode mismatch. On average, we achieve a de-barcoding efficacy of > 90% (Troubleshooting 8).

**CRITICAL:** The de-barcode function in the CyTOF software is based on the previously published Premessa code^43^ and applies only one barcode separation threshold across all pooled samples (https://github.com/ParkerICI/premessa.git) (Figure 8C). This may lead to a high unassigned event count and lower events for downstream data analysis.

### De-barcoding using the CATALYST code in R

Here, we present an alternative approach for de-barcoding the pooled data that is preferably used when the pooled sample contains individual samples with varying cell counts and sample quality. The CATALYST (Cytometry dATa anALYSis Tools) code allows for defining a barcode separation threshold for each sample and ensures a higher de-barcoding efficiency with fewer unassigned events (Figure 8G).^44^

30. De-barcoding using the CATALYST package in R.
  a. Install R and R Studio as well as the packages tidyverse, CATALYST, and flowCore.
  b. Prepare a CSV file containing the sample ID and the respective barcode ID and save it within the folder of the FCS files for the pooled sample.
  c. Run the code below and check the recommended barcode thresholds.

# Install dependancies in R to run debarcoding of pooled .fcs files with CATALYST.

> Sys.setLanguage(lang = “en”)

> if (!require(“BiocManager”))

> install.packages(“BiocManager”)

> BiocManager::install(version = “3.18”)

> BiocManager::install(“CATALYST”)

> BiocManager::install(“flowCore”)

# Load .fcs file and csv. File with debarcoding key.

> Sys.setLanguage(lang = “en”)

> loc <- readline(“Enter the folder location of .fcs ")

> loc <- gsub(“∖∖∖∖”, “/”, loc)

> setwd(loc)

> library(CATALYST)

> bckey <- as.matrix (read.csv(“barcoding key.csv”, row.names = 1, check.names = FALSE ) )

> dr <- list.files(“./”, full.names = TRUE, pattern = “.fcs”)

> dr <- basename(dr)

>cy_dat <- prepData(dr)

>   rownames(cy_dat) <- paste(rowData(cy_dat)$channel_name, rowData(cy_dat)$marker_name, sep = “_”)

> gc()

# Run debarcoding of normalized, pooled sample file and determine cutoff point.

> setwd(loc)

> cy_dat <- assignPrelim(cy_dat, bckey)

> gc()

> cy_dat <- estCutoffs(cy_dat)

> gc()

> unique_bc_ids <- unique(cy_dat$bc_id)

> all_pltyield <- list()

> all_pltyield <- lapply(unique_bc_ids, function(id) {

> plotYields(cy_dat, which = id) +

> ggplot2::theme(legend.position = “right”)

> })

> gc()

# Plot the yield data for each individual sample incl. the respective cutoff.

> dir.create(“Plots”, showWarnings = FALSE)

> for (i in 1:length(all_pltyield)) {

> fl_pth <- file.path(“Plots”, paste0(“YieldPlot”, i, “.png”))

> ggplot2::ggsave(paste0(fl_pth), plot = all_pltyield[[i]], width = 7, height = 4, dpi = 300)

> }

# Apply barcode cutoffs.

> cy_dat <- applyCutoffs(cy_dat)

# Plot total event count per sample.

> x <- matrix(rep(NA, ncol(metadata(cy_dat)$bc_key)), nrow = 1)

> rownames(x) <- “0”

> metadata(cy_dat)$bc_key <- rbind(metadata(cy_dat)$bc_key, x )

> plotevent <- plotEvents(cy_dat, which = “all”, n = 25)

> metadata(cy_dat)$bc_key <- metadata(cy_dat)$bc_key[-which(rownames(metadata(cy_dat)$bc_key) == “0”),]

> for (i in 1:length(plotevent)) {

> fl_pth <- file.path(“Plots”, paste0(“plotevent”, i, “.png”))

> ggplot2::ggsave(fl_pth, plot = plotevent[[i]], width = 7, height = 4, dpi = 300)

> }

> gc()

> bc_chs <- rowData(cy_dat)$channel_name[which(rowData(cy_dat)$is_bc)]

# Plot Mahalanobis distance.

> antigen <- rownames(cy_dat)

> rownames(cy_dat) <- channels(cy_dat)

> mahal <- list()

> unique_bc_ids <- unique_bc_ids[unique_bc_ids != 0]

> for (i in 1:length(unique_bc_ids)) {

> mahalplot <- plotMahal(cy_dat, which = unique_bc_ids[i])

> fl_pth <- file.path(“Plots”, paste0(“plotmahal”, i, “.png”))

> ggplot2::ggsave(fl_pth, plot = mahalplot, width = 7, height = 4, dpi = 300)

> }

> rownames(cy_dat) <- antigen

> gc()

# Plot cell count per sample.

> counts <- table(colData(cy_dat)$bc_id)

> png(paste0(loc,"/Plots/barplot_counts.png”), width = 800, height = 600)

> barplot(unname(counts), names.arg = names(counts), col = “skyblue”,

  main = “Events Counts”, xlab = “Barcode ID”, ylab = “Counts”)

> dev.off()

> gc()

# Save individual, debarcoded .fcs files for each sample within the pool.

> library(flowCore)

> cy_dat <- cy_dat[, cy_dat$bc_id != 0]

> (fs <- sce2fcs(cy_dat, split_by = “bc_id”, keep_cd = TRUE))

> all(c(fsApply(fs, nrow)) == table(cy_dat$bc_id))

> ids <- fsApply(fs, identifier)

> dir.create(“Debarcoded”, showWarnings = FALSE)

> for (id in ids) {

 ff <- fs[[id]]

 fn <- sprintf(“sample_%s.fcs”, id)

 fn <- file.path(“Debarcoded”, fn)

 write.FCS(ff, fn)

> }

> gc()

### Data pre-processing and quality control

**Timing: 2 h**

**CRITICAL:** A hyperbolic arcsine (arcsinh) transformation with a cofactor of 5 is commonly applied for mass cytometry data. However, the cofactor can be adjusted depending on the characteristics of certain datasets. This transformation is already implemented in the obtained FCS files from the CyTOF (Figure S5C, troubleshooting 9).

In this section, we explain the data pre-processing and quality control steps that are necessary before data analysis, including the removal of unspecific signals, e.g., originating from ion cloud fusion events, dead cells, debris, or instrument fluctuations from each FCS file.

**Figure 9:**
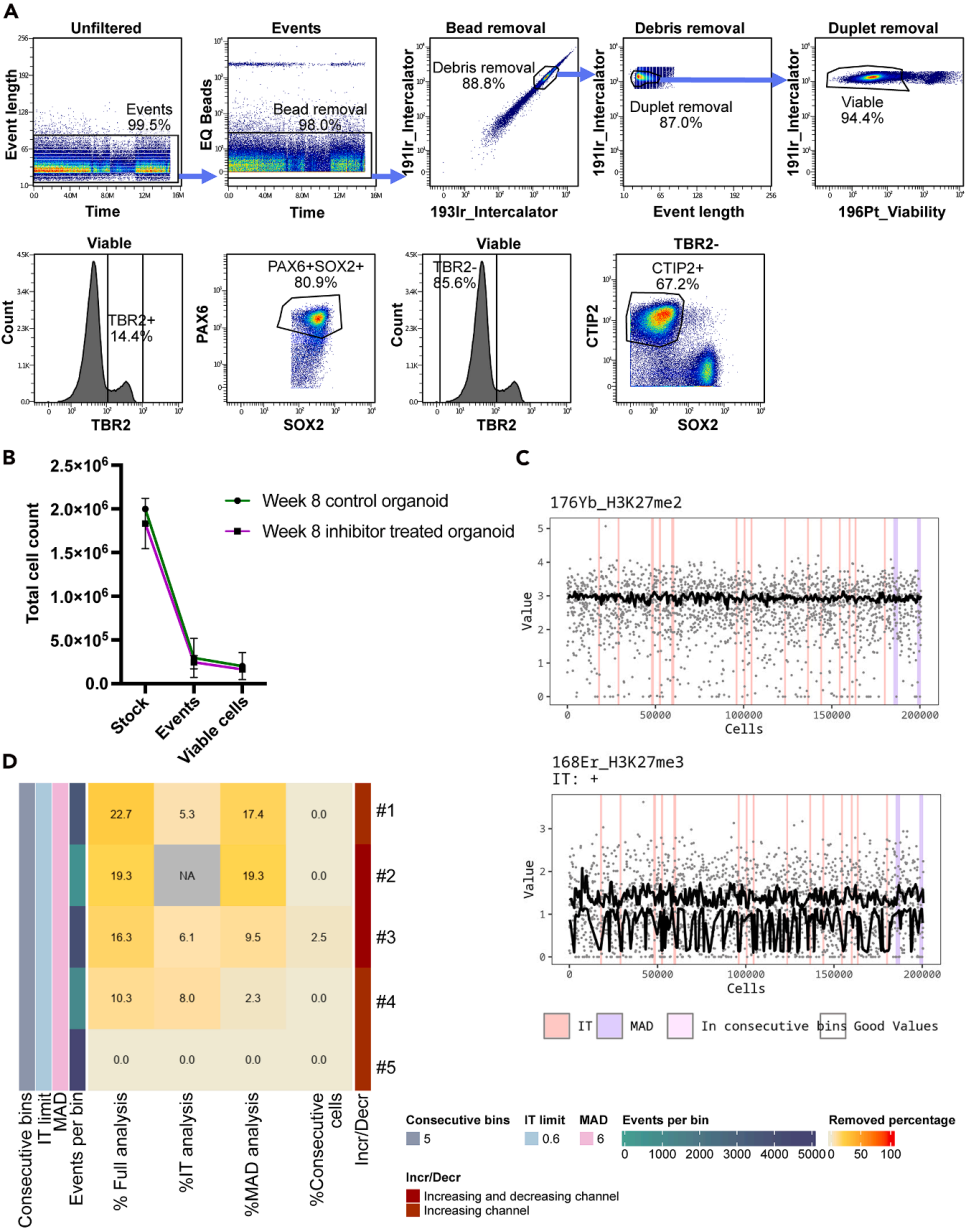
Data preprocessing and clean-up for downstream data analysis using OMIQ software (A) Gating workflow from unfiltered events detected by the machine to viable population, which is further divided into cell-type marker-specific cell populations. The first plot shows the selection of events, followed by the removal of beads, debris, doublets, and dead cells. Polygon selected areas are the populations of interest for each step. (B) Line plot showing the number of cells used for staining, number of events detected by CyTOF machine after staining, and the count of viable cells that are analyzed in the downstream analyses, for week-8 control organoids or methyltransferase inhibitor-treated cortical organoids (n=3 for each group, error bars represent standard deviation). (C) Automated data cleanup performed with the PeacoQC) algorithm. The algorithm determines density peaks per channel and removes low-quality events based on their position in the isolation tree (IT) and on their maximum Mahalanobis Distance (MAD) from the density peaks. Therefore, the algorithm combines events over time in defined bins. The percentage of consecutive cells gives a value when small bins are removed around bin areas that were removed based on IT and MAD. The marked data points are detected as fluctuations or outliers and are removed from the.fcs files. Incr/Decr: increasing/decreasing markers over the time of measurements. (D) Example of a PeacoQC result summary for 5 samples that were processed together (see also Figure S5) (related to Steps 31 to 33).

***Note:*** The unwanted events can be removed either through manual gating of the Gaussian parameter and the automated cleanup tool PeacoQC with the cloud-based software OMIQ (Dotmatics) (Figure 9).

***Note:*** High-quality data can only be obtained with high-quality samples. Therefore, we highly recommend improving sample preparation and staining before extending data clean-up and normalization.

31. Data scaling to adjust the mean metal intensity range in the cloud-based software OMIQ.
  a. Create a new data set and upload all FCS files to OMIQ.
  b. Create a new workflow and select the root task “Scaling”.
  c. Adjust min and max values for each marker separately so that all events are displayed.
32. Remove unspecific signals by manual gating of the Gaussian parameter.
  a. Select the task gating in the data analysis workflow.
  b. Show all events from one sample as a dot plot with the time parameter on the X-axis and the event length on the Y-axis.
  c. Draw a gate around the population with an event length between 10 and 100 (Figure 9A).
  d. Double-click within the selected area to subsequently gate further downstream in the hierarchy.
  e. Remove the EQ6 Beads with a Y-axis gate for ^140^cerium (^140^Ce) along the time at the X-axis.
  f. Remove ion cloud fusion events by subsequent gating for the parameter center, width, residual, offset, and amplitude at the Y-axis along the time at the X-axis.
  g. Define cellular events based on the Cell-ID signal of ^191^iridium (^191^Ir).
    i. Choose first the ^191^iridium positive population on the Y-axis along the time at the X-axis.
    ii. Subsequently, check the co-signaling of ^193^iridium versus ^191^iridium.
  h. To remove doublets, draw a polygon gate around the population within the ^191^iridium versus event length blot and exclude the ^191^iridium-high, event length high events.
  i. Define events of viable cells by gating for ^191^iridium-positive and ^196^platinum (^196^Pt)-negative events.

***Note:*** Avoid stringent gating for the Cell-ID population because different cells in a heterogeneous sample may differ in the amount of DNA.

**CRITICAL:** Perform a data quality check for signal spillovers by generating the NxN matrix in the figure task with OMIQ. In case signal spillover is observed, a compensation matrix can be applied to correct the data with the CATALYST package.

33. Automated data clean-up with the PeacoQC (Peak Extraction and Cleaning Oriented Quality Control) algorithm.
  a. Open the subsequent task PeacoQC within the data workflow.
  b. Select all files and features.
  c. Run the algorithm with a maximum allowed median absolute deviation (MAD) of 6, an isolation tree (IT) gain limit of 0.6, and a minimum number of consecutive bins of 5.
  d. Check the obtained reports for each sample and the cohort to ensure that the removed events are correct (Figures 9B and 9C).
  e. Open the subsequent gating task and draw a quadrant gate around the PeacoQC-passed events.
  f. Select the subsequent task subsampling and define a unified event count for all samples that should be analyzed together.

### Dimensionality reduction and cluster analysis with the cloud-based software OMIQ

**Timing: Variable**

**CRITICAL:** It is essential to have a clear understanding of the biological material being analyzed to optimize gating strategies and interpret the obtained results from cluster analysis. It is important to understand the selective expression of the cell lineage markers across different cell populations to interpret their distribution within the obtained cell clusters.

This section explains how to get started with high-dimensional mass cytometry data analysis. In our study, we analyzed the Epi-CyTOF data from cortical organoids using the cloud-based software OMIQ. As a starting point, we recommend the Cytometry Analysis Tutorial (https://www.omiq.ai/guides/cytometry-analysis-tutorial).

**Figure 10:**
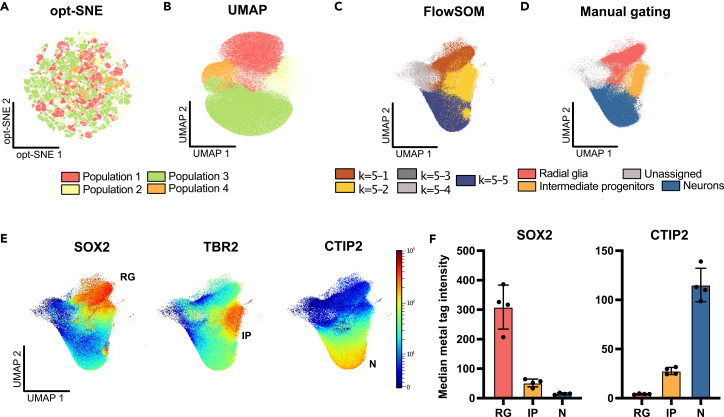
Dimensionality reduction, clustering, and validation of cell type clusters using OMIQ software (A and B) Dimensionality reduction of an example dataset using opt-SNE (A) or UMAP (B) displays distinct cell populations in different colors. Compared with opt-SNE, UMAP better preserves the global data structure, providing a better representation of the cellular composition. (C) Clustering by the FlowSOM algorithm with a manually selected comma-separated *k* value of 5. Clusters 1 to 5 are shown in different colors. (D) Cell clusters that are assigned according to prior gating strategy are shown in orange for SOX2^+^PAX6^+^ radial glia (RG), yellow for TBR2^+^ intermediate progenitors (IP), and blue for CTIP2^+^ neurons (N) in week-8-old cortical organoids. The gray population is unassigned viable events. (E) Color-continuous UMAP plots of cell type markers SOX2 (RG), TBR2 (IP), and CTIP2 (N), validating distinct cell clusters shown in (C and D). (F) Bar plots showing the median metal tag intensities for cell type markers PAX6 (RG) and HuCD (N), further confirming the cell type clustering shown in (C-E). Error bars represent the standard deviation of 4 replicates, bar plots represent the mean values (related to Steps 34 to 38).

***Note:*** Several dimensionality reduction algorithms, such as principal component analysis (PCA), optimized t-distributed stochastic neighbor embedding (opt-SNE), or uniform manifold approximation and projection (UMAP), help to visualize the obtained Epi-CyTOF data. We highly recommend trying two to three algorithms for each data set to determine the best-suited one that allows for illustrating the biological differences between different sample subsets (Figures 10A and 10B). A manual gating strategy should be used additionally to validate the obtained findings (troubleshooting 10)**.**

34. Dimensionality reduction using the UMAP algorithm in OMIQ.
  a. Open the UMAP task subsequently to subsampling.
  b. Select all data files and all lineage markers as features.
  c. Run UMAP with the settings of 15 neighbors and a minimum distance of 0.4.
  d. Check the obtained clustering within the subsequent task for figures. Eventually, adjust the UMAP setting and re-run the analysis.
  e. Visualize all markers within a color-continuous scatterplot and inspect the expression pattern of all markers across the obtained clusters for unspecific and missing signals.

***Note:*** The setting “Neighbors” controls the balance between local and global data structure, where smaller values emphasize local relationships and larger values capture broader patterns. The “Minimum distance” determines how closely the points within clusters are packed. Lower values produce denser clusters, while higher values yield more spread-out embeddings. The “Euclidean” distance is the most commonly used and recommended option. “Random seed” can be set to any number to control the reproducibility of the results.

**CRITICAL:** It is crucial to visualize all viable events to prevent artifactual cluster formation, e.g., originating from debris. When using color-continuous scatterplots, ensure that the minimum and maximum values of the color scale are appropriately adjusted to avoid misleading interpretations of marker expression.

35. Cluster analysis using the Flow Self-Organizing Map (FlowSOM) algorithm.
  a. Select the task from “Clustering” and choose FlowSOM.
  b. Adjust the settings “xdim” and “ydim” to define the total number of SOM nodes. For initial analysis, values between 5 and 15 are recommended.
  c. Enter comma-separated *k* values representing the expected number of clusters based on the biological data and click “Run FlowSOM”.
  d. Open the downstream task “Gating”, add categorical filters, and name the obtained metaclusters.

***Note:*** Several clustering algorithms are available in OMIQ, such as Phenograph and Spanning-tree Progression Analysis of Density-normalized Events (SPADE). SPADE maps density-normalized events via minimum spanning trees, whereas Phenograph uses k-nearest neighbor graphs and Louvain communities to identify clusters. For CyTOF data, FlowSOM is the most widely used algorithm for hierarchical clustering because of its fast and robust performance.^45^ We recommend trying out different approaches.

36. Cluster validation using manual gating.
  a. To identify the cellular identity of the metaclusters, overlay UMAPs with manual gating (Figures 10C and 10D).
  b. Examine the abundance patterns of main cell type markers within the clusters using color-continuous UMAP illustrations (Figures 10E and 10F).
  c. If there are discrepancies between manual gating and unsupervised clustering, repeat the analysis by adjusting *k* values or merging clusters.
  d. Revisit the manual gating strategy to ensure that unwanted events are excluded from the analysis.
37. Statistical analysis of marker abundance and median metal tag intensities.
  a. Visualize the Epi-CyTOF data using UMAPs and plot the functional markers (Step 37).
  b. Export median metal tag intensities as.csv file using the “Export Stats” function in OMIQ.
  c. Perform statistical tests on median intensity values to identify significant differences across distinct cell clusters.
  d. Use heatmaps to check all functional markers, separated by replicates, to assess reproducibility.^1^

***Note:*** The cloud-based software OMIQ provides several statistical tools for group comparison. In our study, we were interested in dissecting differences in epigenomic states and profiles across clusters within the same sample.

**CRITICAL:** Within this study, we used four technical replicates per cell line to ensure robust and reproducible data analysis using group comparison.

38. Export of data for cell type annotation and statistical analysis.
  a. Perform data cleanup in OMIQ as shown in Figure 9A.
  b. Export viable cell count as FCS Files for each sample.

### Quantification and statistical analysis with R

**Timing: Variable**

***Note:*** All statistical analyses were performed using R (v4.4.2; R Foundation for Statistical Computing). The Core Bioconductor infrastructure packages were used for data handling, including SingleCellExperiment (v1.28.1),^25^ SummarizedExperiment (v1.36.0),^26^ S4Vectors (v0.44.0),^27^ and Biobase (v2.66.0).^28^

**Figure 11:**
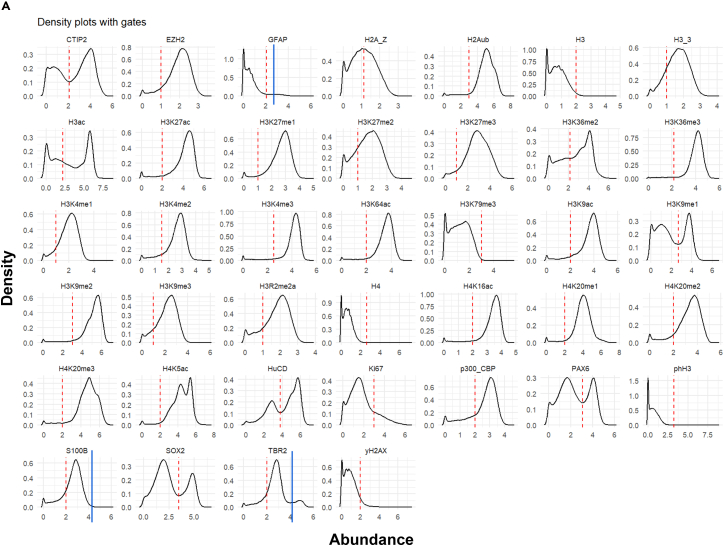
Density plots showing the intensity level of all Epi-CyTOF markers to define thresholds to discriminate cell populations with positive from negative expression Histograms showing the abundance of each channel with an automatically detected threshold (dotted, red line) separating positive and negative populations. For some of the cell type markers, these thresholds were adjusted manually (blue lines) according to biological expectations regarding the presence of certain cell clusters.

In this section, we present an R script that uses the cleaned mass cytometry data exported from OMIQ for clustering and cell type annotation. Unlike OMIQ, which provides a predefined user-friendly platform, analysis performed in the open-source programming environment R offers flexibility, allowing customization and integration of bioinformatic tools while yielding comparable metacluster results as obtained with OMIQ (Figure 11). All scripts and code required to reproduce the analysis are available in the paper-specific GitHub repository (https://github.com/Stacheltier/EPI-CyTOF). The computational environment and package versions are documented to ensure reproducibility.

**CRITICAL:** OMIQ-exported viable events from mass cytometry data were imported from cleaned FCS files (FlowRepository ID: FR-FCM-Z8DC) using the flowCore package (v2.18.0, https://doi.org/10.18129/B9.bioc.flowCore).^21^^,^^46^ Preprocessing and data organization were performed using the CATALYST framework (v1.30.29, https://doi.org/10.18129/B9.bioc.flowCore).^22^^,^^23^^,^^47^

39. Import of OMIQ-derived, cleaned mass cytometry data.
  a. Import FCS files using read.flowSet(., transformation = FALSE, truncate_max_range = FALSE) from the flowCore package.
  b. Import metadata containing sample information and panel data containing marker–channel information using read.csv().
  c. Generate a *SingleCellExperiment* object using prepData() from the CATALYST package.
  d. Rename assay(“counts”) to assay(“exprs”) as needed for subsequent analytical steps.

# Import FCS files.

fcs_fowset<-read.flowSet(path="∼/data", transformation=FALSE, truncate_max_range=FALSE)

# Import metadata and panel information.

md <- read.csv("metadata.csv", sep = ";")

panel<-read.csv("panel.csv", sep = ";")

# Generate SingleCellExperiment object

sce<-CATALYST::prepData(fcs_flowset,transform=FALSE,features=NULL,

md=md,panel=panel,panel_cols = list(channel = "channel_name",antigen = "marker_name",class = "marker_class"))

assay(sce, "exprs") <- assay(sce, "counts")

assays(sce)$counts <- NULL

**CRITICAL:** The neural lineage markers SOX2, CTIP2, TBR2, and PAX6 were used for clustering and cell type annotation. FlowSOM clustering was performed on the full dataset using a self-organizing map with a 30 × 30 grid and 10 metaclusters. The gates to define positive or negative marker expression were set manually for each marker on arcsinh-transformed expression values (cofactor = 5) using a representative subset of 50,000 randomly selected events (Figure 11).

***Note:*** Clusters were classified as neurons (N) by positive CTIP2 expression the absence of SOX2, TBR2, and PAX6. Intermediate progenitor cells (IP) were defined by positive TBR2 expression. Radial glia (RG) cells were identified by co-expression of SOX2 and PAX6 and the absence of CTIP2 and TBR2. Cells that did not meet these criteria were labeled as unassigned events. The unassigned events were iteratively re-clustered three times using identical FlowSOM parameters and reassigned based on the same marker-based classification rules.

40. Clustering and cell type annotation.
  a. Extract assay(“exprs”) of 50,000 randomly selected events to generate a pivot table of expression values using sce_to_long_df() function.
  b. Calculate density curves for each marker using the density() function from the stats package and plot the curves using ggplot() from the ggplot2 package combining publication specific functions check_marker_modality(), compute_all_gates() and plot_gates_density().
  c. Apply cluster(sce, features = “type”, xdim = 30, ydim = 30, maxK = 10) from the CATALYST package to perform FlowSOM clustering on the full dataset.
  d. Plot marker expression of the generated clusters using plotExprHeatmap() and plotPbExprs() from CATALYST.
  e. Set gates manually based on expert knowledge and assign cell types using the mergeClusters() function from CATALYST.
  f. Subset unassigned events into a separate SingleCellExperiment object.
  g. Repeat steps c–f three times to iteratively refine clustering and cell type annotation of unassigned events.
  h. Merge the generated SingleCellExperiment objects into a single SingleCellExperiment object to obtain a fully annotated dataset for downstream analyses.

**Figure 12:**
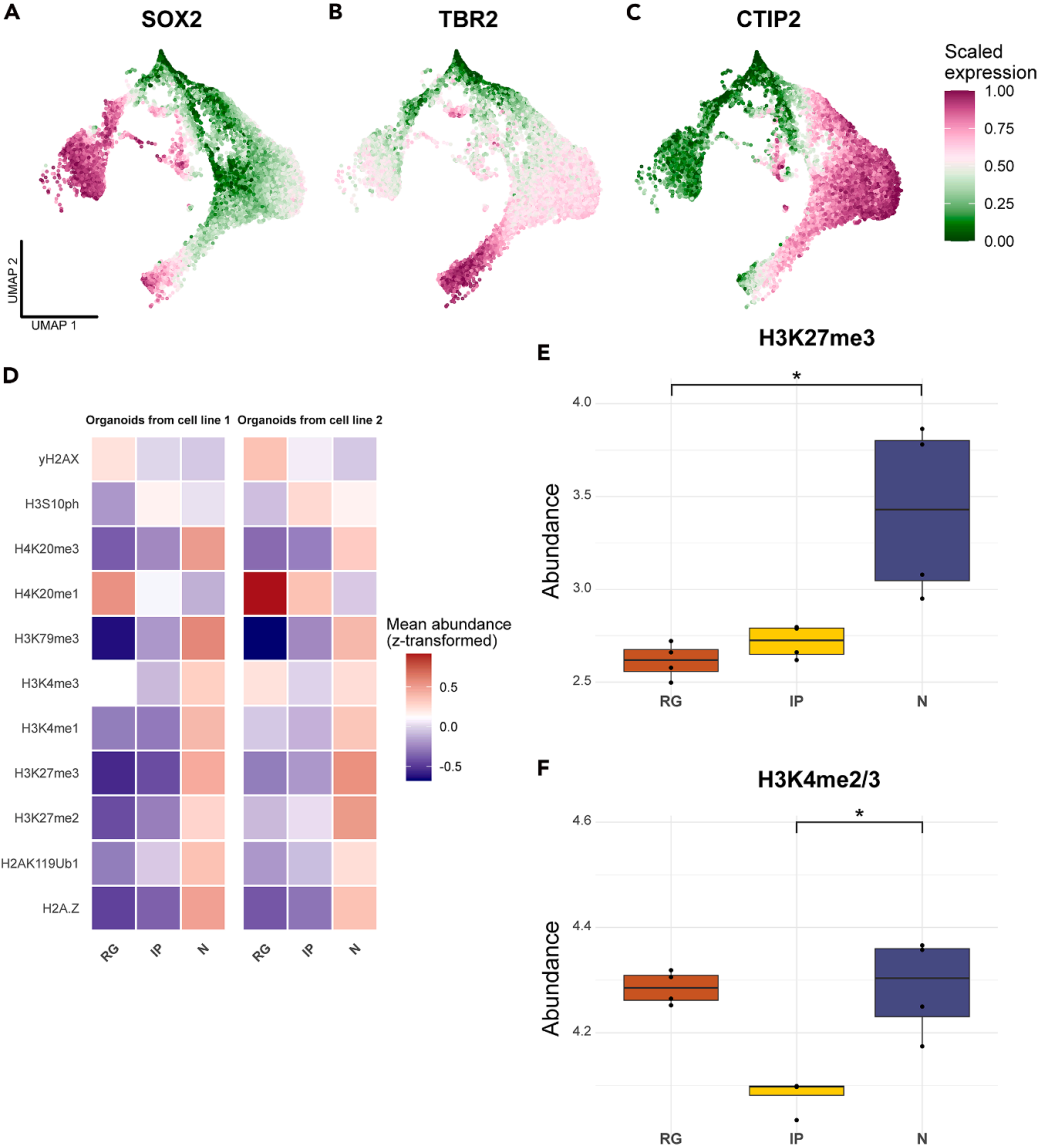
Data analysis for Epi-CyTOF using a customized R-based analysis pipeline (A–C) UMAP plots display the expression of the three lineage-defining markers SOX2 (A), TBR2 (B), and CTIP2 (C) used for cell-type annotation in two cortical organoid cell lines C1 (left) and C2 (right). Color intensity represents relative marker abundance (red = high, green = low). Marker expression was scaled per plot to enhance contrast. (D) Heatmaps show the median metal intensity for histone modifications (rows) displaying significant differences across the three cell types: radial glia (RG), intermediate progenitors (IP), and differentiated neurons (N). The color scale represents mean abundance intensities after z-transformation (blue = low, white = intermediate, red = high). (E and F) Boxplots depict the mean abundance values of the histone 3 lysine 27 trimethylation (H3K27me3) (E) and histone 3 lysine 4 di- and trimethylation (H3K4me2/3) (F) in cortical organoids stratified into the three cell types: radial glia (RG), intermediate progenitors (IP), and differentiated neurons (N). For each marker, individual sample means are shown as black dots overlaid on the boxplots (related to Steps 39 to 41).

# Clustering and cell type annotation

## Step 43a

df_long <- sce_to_long_df(sce, assay_name = "exprs", marker_col = "marker_name", max_cells_per_marker = 50000, seed = 123)

## Step 43b

modality_df <- check_marker_modality(df_long)

gates_df <- compute_all_gates(df_long, modality_df, lower = 1, upper = 4, method_unimodal = "quantile", quantile_val = 0.05)

plot_gates_density(df_long, gates_df)

## Step 43c

csce <- cluster(sce, features = "type", xdim = 30, ydim = 30, maxK = 10)

## Step 43d

plotExprHeatmap(sce, k="meta10")

plotPbExprs(sce, k="meta10")

## Step 43e

merging_table<-read.csv("merging.csv", sep = ";")

sce<-mergeClusters(sce,k="emata10", id="cell_types", table = merging_table)

## Step 43f

is_undefined <- colData(sce)$cell_type == "undefined"

sce_undefined <- sce[, is_undefined]

***Note:*** Dimensionality reduction was performed using UMAP to visualize cellular heterogeneity with the CATALYST package. Only markers classified as cell–type–defining features were included. The resulting low-dimensional representations were used exclusively for visualization and were not applied for lineage-based downstream analyses or statistical analysis (Figures 12A–12C).

### Statistical analysis for cluster comparison with R

This section includes and guides you through an R script aims to perform statistical analysis for cluster comparison within the CyTOF data.

**Timing: Variable**

41. Statistical analysis of cell type-specific expression for histone modification markers and epigenetically active enzymes.
  a. Extract the expression values for the state markers using the command assay(“exprs”) and generate a data frame with mean expression values per cell type and organoid in pivot table format.
  b. Perform statistical tests to identify markers with significantly different expression between different neural lineages using kruskal.test(), followed by Dunn’s post-hoc tests with Benjamini–Hochberg correction using the command dunnTest() from FSA code.
  c. Generate plots using ggplot() and annotate significance (Figures 12D and 12E).

# Compute UMAP embeddings.

CATALYST::runDR(sce, dr = c("UMAP"), features = "type", assay = "exprs", cells = 5000)

# Visualize UMAP representations

CATALYST::plotDR()

# Statistical Analysis of cell type specific expression

## Step 44a

state_markers_vec <- state_markers(sce)

pb_df <- assay(sce, "exprs")[state_markers_vec, ] %>%

 t() %>%

 as.data.frame() %>%

 cbind(as.data.frame(colData(sce)[, c("sample_id", "cell_type", "organoid")])) %>%

group_by(sample_id, cell_type, organoid) %>%

 summarise(across(all_of(state_markers_vec), mean, na.rm = TRUE), .groups = "drop")

## Step 44b

num_markers <- markers[sapply(pb_df[markers], is.numeric)]

results <- list()

for (org in organoids) {

 df_org <- pb_df %>% filter(organoid == org)

 for (m in num_markers) {

 df_m <- df_org %>%

  dplyr::select(cell_type, all_of(m)) %>%

  rename(value = !!m) %>%

  filter(!is.na(value)) %>%

  mutate(value = as.numeric(value))

  group_counts <- df_m %>% count(cell_type)

 if (nrow(group_counts) < 2 || any(group_counts$n < 2)) next

# Kruskal-Wallis-Test

kw <- kruskal.test(value ∼ cell_type, data = df_m)

# Dunn-Test (Benjamini-Hochberg)

 dunn_res <- FSA::dunnTest(value ∼ cell_type, data = df_m, method = "bh")$res

   results[[paste(org, m, sep = "_")]] <- list(

   organoid = org,

   marker = m,

   kruskal = kw,

   dunn = dunn_res

  )

 }

}

kruskal_df <- purrr::map_dfr(results, function(x) {

 broom::tidy(x$kruskal) %>%

  mutate(

  organoid = x$organoid,

  marker = x$marker

  )

})

## Expected outcomes

Using this Epi-CyTOF protocol, we were able to perform epigenetic profiling at the single-cell level using tissue and organoid samples by integrating specific cell-type markers. Epi-CyTOF detects changes in the abundance of epigenetic markers across different cell types within one sample as well as between different samples. With an appropriate implementation of other cell type-specific markers, Epi-CyTOF can also be applied to other sample types and sources.

To set up the Epi-CyTOF procedure, including the experimental procedures, antibody testing, and the data analysis workflow, we required approximately one year. If a CyTOF instrument is not available locally, samples can be stained, fixed, and shipped for service measurements to dedicated shared resource laboratories. When planning mass cytometry measurements, we recommend contacting the experienced local scientist before detailed project planning to obtain advice. Useful tips and tricks can be additionally found at the cytoforum (https://cytoforum.stanford.edu/).

Before the actual experiments, the antibodies have to be titrated with the sample types to be used in the main experiment. It is also important to test the antibody specificity with positive and negative controls, especially if the antibodies have not been tested for CyTOF applications (Figures 4 and 5).

We established the Epi-CyTOF staining for human cortical organoids using ten neural cell type markers to differentiate between the neural cell lineages, radial glia, intermediate progenitor cells, and neurons.^1^ Across the different neural cell populations, a variety of repressive and activating histone modifications are analyzed to investigate the epigenetic state at the single-cell level. Before applying the Epi-CyTOF panel for epigenetic profiling in cortical organoids, the neuronal lineage markers are selected by their performance in conventional flow cytometry and are validated on primary fetal brain tissue.^1^ The epigenetic markers are validated following small molecule inhibition targeting the embryonic ectoderm development (EED) protein, which is part of the polycomb repressive complex 2 (PRC2). As previously published, the reduced activity of the histone methyltransferase complex leads to a reduced tri-methylation of histone 3 at lysine residue 27, and validated the anti-H3K27me3 antibody.^1^

As a next step, we adjusted the panel for the staining of gastro-esophageal tumoroids. For antibody specificity testing, the expression of gastrointestinal lineage markers mucin 5AC (MUC5AC), mucin 6 (MUC6), chromogranin-A (CHGA; also known as parathyroid secretory protein 1), and cadherin 1 is confirmed using normal stomach tissue and ovarian tumor-derived organoids as positive control, as well as cortical organoids as negative control (Figure 5A). The histone acetylation is validated using gastro-esophageal tumoroids treated for 4 and 12 h with the histone deacetylase (HDAC) inhibitor Panobinostat^48^ (Figure 5B).

In this protocol, we present two different options for barcoding: Pd-based for fixed samples and TeMal-based for viable samples (Figure 8). Finally, we illustrate the use of several algorithms for data pre-processing, illustration, and statistics, either using the cloud-based platform OMIQ or an R-based pipeline (Figures 9, 10, 11, and 12).

*Future perspective:* The mass cytometry approach described in this protocol begins with a single-cell suspension. While this allows obtaining epigenetic information at the single-cell level, this method does not preserve positional information within the tissue context. Further developments of the method include the application of Imaging Mass Cytometry (IMC).^49^^,^^50^ We are currently testing the Epi-CyTOF panel on tissue sections of the cortical organoids for imaging mass cytometry, which in the future may help to preserve spatial information that is often key in complex tissues. Moreover, IMC can be combined with spatial RNA-seq or MALDI imaging analysis,^51^^,^^52^ which further allows direct linking of epigenetic marking with gene expression outcomes. These generated multi-modal data sets require workflows for data integration in order to analyze the molecular profiles within spatially separated regions.^53^^,^^54^

## Quantification and statistical analysis

For the “Statistical analysis for cluster comparison with R” section, all statistical analyses were performed using R (v4.4.2; R Foundation for Statistical Computing) on a Windows 11 x64 platform. Analyses were performed separately for each organoid cell line, taking technical duplicates into account. State marker expression values were aggregated at the sample level by calculating the mean expression per cell type and organoid. Because marker expression data did not meet assumptions of normality and homogeneity of variances, non-parametric methods were applied throughout. Differences between lineage groups were assessed using the Kruskal–Wallis rank-sum test. Only markers with measurements in at least two lineage groups and a minimum of two observations per group were included.

For markers showing a significant overall effect (p < 0.05), pairwise post-hoc comparisons between lineages were performed using Dunn’s test with Benjamini–Hochberg false discovery rate (FDR) correction. Markers with an adjusted p-value < 0.05 were considered statistically significant. Data manipulation and aggregation were performed using tidyverse packages (dplyr v1.1.4 (https://dplyr.tidyverse.org),^30^ tidyr v1.3.1 (https://tidyr.tidyverse.org),^31^ purrr v1.1.0 (https://purrr.tidyverse.org/),^32^ tibble v3.3.0 (https://tibble.tidyverse.org/).^33^ Kruskal–Wallis tests were conducted using base R (stats package), and Dunn’s post-hoc tests using the FSA package (v0.10.0, https://fishr-core-team.github.io/FSA/).^34^ Test results were formatted with the broom package (v1.0.9, https://broom.tidymodels.org/).^35^ Visualizations were generated using ggplot2 (v4.0.1, https://https://ggplot2.tidyverse.org/)^36^ and ggsignif (v0.6.4, https://psyarxiv.com/7awm6).^37^

## Limitations

Although innovative, the Epi-CyTOF method has limitations that need to be considered when. The data quality highly relies on the availability, specificity, and performance of the selected antibodies. We highly recommend testing selected antibodies using positive and negative controls and validating the results with another method, such as immunofluorescence using different antibody clones. The number of markers per panel is currently limited to approximately 45 to 50 due to the lack of availability of stably purified metal isotopes for antibody conjugation, and could be extended by integrating single-cell RNA sequencing data. Performing single-cell RNA sequencing experiments in parallel with subsequent data integration would allow us to obtain novel insights into co-regulatory epigenetic effects on gene and protein expression. Moreover, Epi-CyTOF illustrates global changes in histone modifications on a single-cell level. Therefore, site-specific methods such as ChIP-seq or Cut&Tag should be used to resolve the genome-wide patterns underlying the observed global changes.

As we present the detailed protocol, there are key factors to further consider for advancing Epi-CyTOF studies. For example, within the Epi-CyTOF panel, we identified two antibodies of concern (SATB1/2-^158^Gd, HOP-^155^Gd) and four with suboptimal performance (H3-^115^In, H3ac-^112^Cd, S100-^169^Tm, and H4-^209^Bi), which will be replaced in the follow-up study. After completion of our study, the antibody H3K4me2/3 (clone C42D8) was reported to lack specificity for the tri-methylation state, showing additional detection of di-methylation. Additionally, some antibodies, such as the H3-^115^In (clone D1H2), were discontinued by the company IonPath during the study and were replaced with custom-conjugated antibodies of the same clone.

A minimal infrastructure needed to get started with Mass Cytometry includes access to a CyTOF instrument either in a shared resource laboratory or in a laboratory of a collaboration partner, basic equipment for sample preparation and cell staining, metal-tagged antibodies and conjugation reagents, and access to analysis tools or established data analysis workflows. Compared with spectral flow cytometry, the per-cell marker cost is similar. The advantage of CyTOF is the high reagent and sample stability, which reduces batch effects and effort in multicenter clinical trials.

## Troubleshooting

### Problem 1

Cell clumping during dissociation of cortical organoids.

### Potential solution

The efficacy of cortical organoid dissociation needs to be monitored. If the organoids are not easily turned into single cells, the digestion time, enzyme mixture, or temperature needs to be optimized for each sample. Additionally, if smaller organoids cannot be pipetted well with large tips, narrower tips or a 22-G syringe needle can be used after enzymatic dissociation for additional manual dissociation.

### Problem 2

Variation in tumoroid dissociation time and single-cell output.

### Potential solution

Adapt dissociation times and increase the number of tumoroids depending on organoid morphology (i.e., cystic or dense). Coat pipette tips with 1% BSA if intensive cell loss is observed.

### Problem 3

Instability and signal loss of specific antibody clones and/or antibody-metal combinations.

### Potential solution

In rare cases, we observe low recovery or decreasing signal intensity after conjugation. If this happens, the antibody clone and/or the metal isotope can be changed and should be tested with a sample of known expression for the specific antigen. Issues of signal loss can occur either due to inefficient binding of the metal isotopes to the polymer, reduced binding of the metal-bound polymer to the chelated constant region of the antibody, or reduced binding efficacy, e.g., due to antigen misfolding after sample preparation. Comparing the signal intensity of positive control cells, IgG capture beads, and/or the antibody solution in dilution mode can help (Figure S1).

### Problem 4

Inefficient or problematic cell permeabilization.

### Potential solution

For cell fixation and permeabilization, PFA and methanol are commonly used, respectively. While it is expected that PFA-based cell fixation adversely affects approximately 5% of extracellular epitopes, the methanol-based permeabilization changes the configuration of almost 50% of intracellular antigens and diminishes antibody-based antigen detection. Within the cortical organoid cell suspension, methanol-based permeabilization reduced the detection of several lineage markers and some epigenetic markers (Figure S3). The staining quality was preserved with the commercially available Nuclear Antigen Perm Buffer. Nuclear permeabilization can also be achieved by other detergents such as saponin, Triton X-100, or NP-40 that disrupt the intact cell and nuclear membrane structure, allowing antibody penetration. The concentration and incubation time of the detergent should be optimized to prevent excessive damage to cellular structures and antigen loss. A proper cell fixation before permeabilization must be ensured to preserve cellular structures.

### Problem 5

Expression loss of extracellular antigens.

### Potential solution

Before fixation of cells, it is necessary to evaluate the staining quality before and after PFA fixation. PFA crosslinks proteins and can alter the conformation of target antigens, potentially reducing antibody binding and altering expression patterns, leading to inaccurate results. Before the study started, the percentage of CDH1^+^CDH17^+^ epithelial cells in gastro-esophageal tumoroids were compared between freshly stained and pre-fixed samples. The double-positive population was reduced after fixation compared to freshly stained cells. (Figure S4). Therefore, to minimize this effect, extracellular markers should be stained before PFA fixation.

### Problem 6

Failure of CyTOF tuning and cell calibration.

### Potential solution

Without a proper CyTOF instrument calibration, we will not start sample measurements. When instrument parameters are detected out of range. We clean the sample lines and re-run tuning with freshly prepared EQ6 beads. Regular instrument clean procedures, e.g., the tubing system once per week or the cones when the detector voltage (DV) increases, prevent tuning failures. We schedule a preventive maintenance annually by a specialized service engineer to ensure long-term stability.

### Problem 7

Nebulizer clogging and increased argon gas pressure.

### Potential solution

Following the recommendations for cell fixation and centrifugation is important to ensure high-quality samples and stable CyTOF measurements. Low-quality samples tend to clog the nebulizer line, leading to disturbances during the measurements. When the nebulizer gas pressure increases and a clog occurs, the measurements have to be stopped, the nebulizer and sample lines removed, cleaned, and replaced. This prolongs the total time of measurements and reduces the recovery of viable cell events per sample.

### Problem 8

Barcode mismatch.

### Potential solution

It is essential to implement quality control documentation to ensure the correct barcode key for individual samples within a pool. A failed de-barcoding can be noticed when the yield plot is negative.

### Problem 9

Data scaling.

### Potential solution

CyTOF data have to be transformed with arcsinh and a cofactor of 5. Changing the co-factor leads to over- or under-compression of signal intensities and modifies the downstream data visualization, clustering, and statistical analysis (Figures S5A–S5C).

### Problem 10

Inadequate separation of cell populations.

### Potential solution

If certain cell populations that are supposed to be distinct are not well-separated, adjust “neighbor” or ”minimum distance”.

## Resource availability

### Lead contact

Further information and requests for resources and reagents should be directed to and will be fulfilled by the lead contact, Claudia Peitzsch (claudia.peitzsch@tu-dresden.de).

### Technical contact

Technical questions on executing this protocol should be directed to and will be answered by the technical contacts, Ezgi Senoglu (ezgi.senoglu@tu-dresden.de) and Vivian Mittné (vivian.mittne@ukdd.de).

### Materials availability

This protocol does not contain newly generated materials.

### Data and code availability

The Epi-CyTOF data for the cortical organoids are available in the FlowRepository: FR-FCM-Z8DC. The data analysis workflow is available within the paper-specific GitHub repository (https://github.com/Stacheltier/EPI-CyTOF).^55^

## Acknowledgments

The mass cytometry-related work was supported by the staff of the Mass Cytometry (CyTOF) Core Facility at the Center of Regenerative Therapies Dresden (10.13039/501100007491CRTD, 10.13039/501100002957TU Dresden, Germany, RRID: SCR_027145). Ezio Bonifacio (10.13039/501100007491CRTD and 10.13039/100031584German Center for Diabetes Research, 10.13039/100031584DZD) raised funding from the 10.13039/501100001659German Research Foundation (10.13039/501100001659Deutsche Forschungsgemeinschaft, 10.13039/501100001659DFG; project no. FZT 111) in 2012 to purchase the suspension mass cytometer CyTOF 2 (Fluidigm). In 2023, he obtained funding from the Federal Ministry for Research, Technology, and Aeronautics (Bundesministerium für Bildung und Forschung, 10.13039/501100002347BMBF) and, together with the Medical Faculty Carl Gustav Carus, TU Dresden, and the Staatsministerium für Wissenschaft, Kultur und Tourismus (10.13039/501100006114SMWK), Germany, purchased the suspension mass cytometer CyTOF XT (Standard BioTools). We would like to thank Parham Pourabbastahvildar for his help in setting up the debarcoding with CATALYST; the effective exchange within the German Mass Cytometry Community (GerMaNet); and Mike Leipold (Stanford University) for the CytoForum as a platform, a valuable source for troubleshooting (https://cytoforum.stanford.edu/viewtopic.php?f=7&t=165). M.A. acknowledges funding from the Center for Regenerative Therapies TU Dresden (CRTD), the 10.13039/501100001659DFG (Emmy Noether Programme, AL 2231/1-1; SPP EPIADAPT 563285578, AL 2231/4-1; and AL 2231/3-1) and ERA-NET Neuron (MEPIcephaly; Federal Ministry of Education and Research (10.13039/501100002347BMBF, 01EW2208). M.A. and E.S. acknowledge the Fritz Thyssen Foundation. F.B., A.E., C.P., and M.A. acknowledge support from the German Centers for Health Research (DZG). The graphical illustrations were created with BioRender.com.

## Author contributions

Writing – original draft, E.S., V.M., and C.P.; writing – review and editing, S.D., A.E., F.B., S.T., and M.A.; methodology, E.S., V.M., S.D., A.E., S.T., and C.P.; supervision, F.B., M.A., and C.P.; project administration, C.P.; funding acquisition, F.B., A.E., C.P., and M.A.

## Declaration of interests

The authors declare no competing interests.

## Declaration of generative AI and AI-assisted technologies in the writing process

During the preparation of this work, the authors used Grammarly for spelling and grammar checks. After using the tool, the authors reviewed and edited the content as needed and took full responsibility for the content of the publication.
