# Supplementary material for: Protocol for single-cell epigenetic profiling in human organoids and tumoroids with Epi-CyTOF
Source: STAR Protoc. 2026 Jul 31;7(3):104695. doi: 10.1016/j.xpro.2026.104695 (PMC13453457; doi:10.1016/j.xpro.2026.104695)
Supplement: Supplementary file 1 [file mmc1.pdf]

Supplementary Information

Supplementary Figures

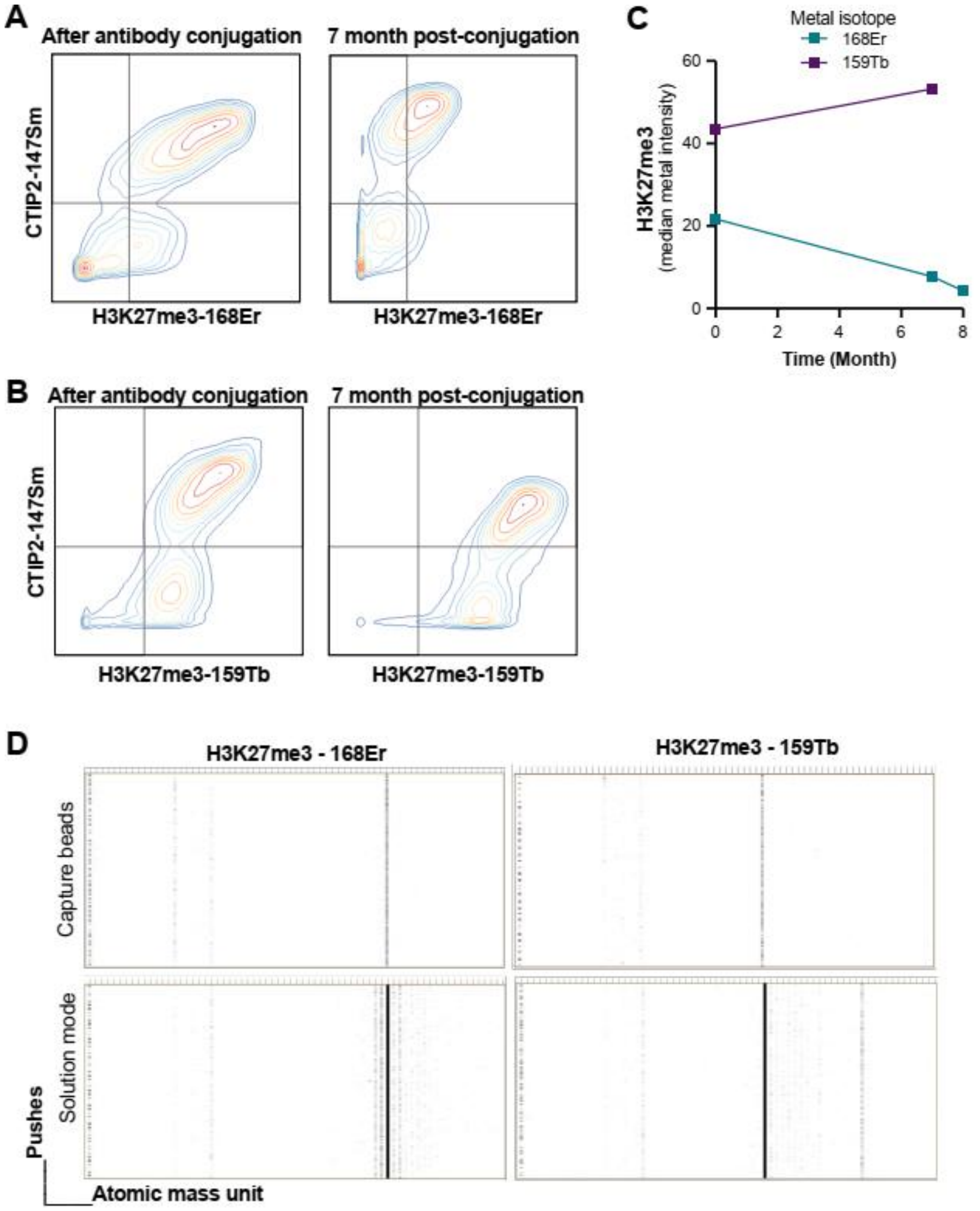

Supplementary Figure S1. Loss of anti-H3K27me3-159Tb signal intensity over time. A) Two-dimensional contour plots illustrate the loss of H3K27me3-^{168}erbium (^{168}Er) signal seven months post-conjugation in a single-cell suspension of cortical organoids. The lineage marker COUP-TF-interacting protein 2 (CTIP2) is a transcriptional regulator that is highly expressed in differentiated neurons and has been used to determine cell identity. The signal loss of the anti-H3K27me3-168Er antibody is illustrated by the reduced signal intensity at 7 months post-conjugation (stock: 0.5 mg/mL, 1:200 dilution, final concentration: 2.5 µg/mL). **B)** The same antibody clone is conjugated with another metal isotope, ^{159}Tb, and the positive signal after 7-month storage at 4 °C is comparable to the test staining directly after conjugation. **C)** The median metal intensity (MMI) of the anti-H3K27me3 antibody conjugated with the isotope ^{159}Tb was higher and stable over time in dissociated cortical organoids compared to the ^{168}Er conjugation (n = 1, each). **D)** Quality control of both custom conjugated antibodies after staining capture beads and measuring the 0.5 mg/mL concentrated antibody solution at the CyTOF XT in dilution mode (**related to Steps 1-3**).

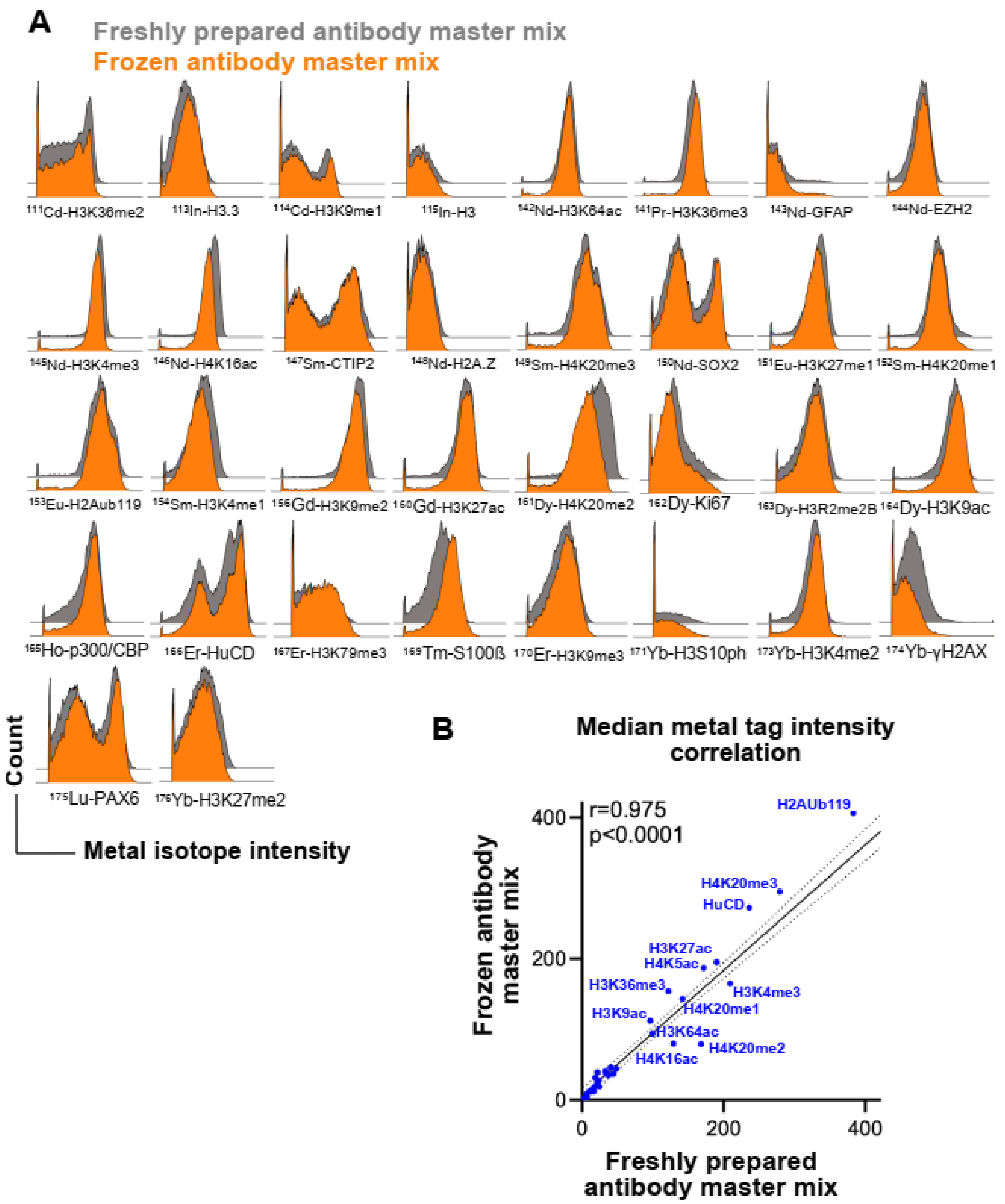

Supplementary Figure S2. Quality control to validate the performance of the frozen antibody master mix. A) Overlay histograms illustrate the metal intensities after staining a single-cell suspension of cortical organoids with freshly prepared versus frozen antibody master mix. **B)** Correlation of median metal intensities for the Epi-CyTOF markers stained either freshly or as frozen master mix (n = 1, Pearson correlation, simple linear regression including 95% CI) (**related to Step 11)**.

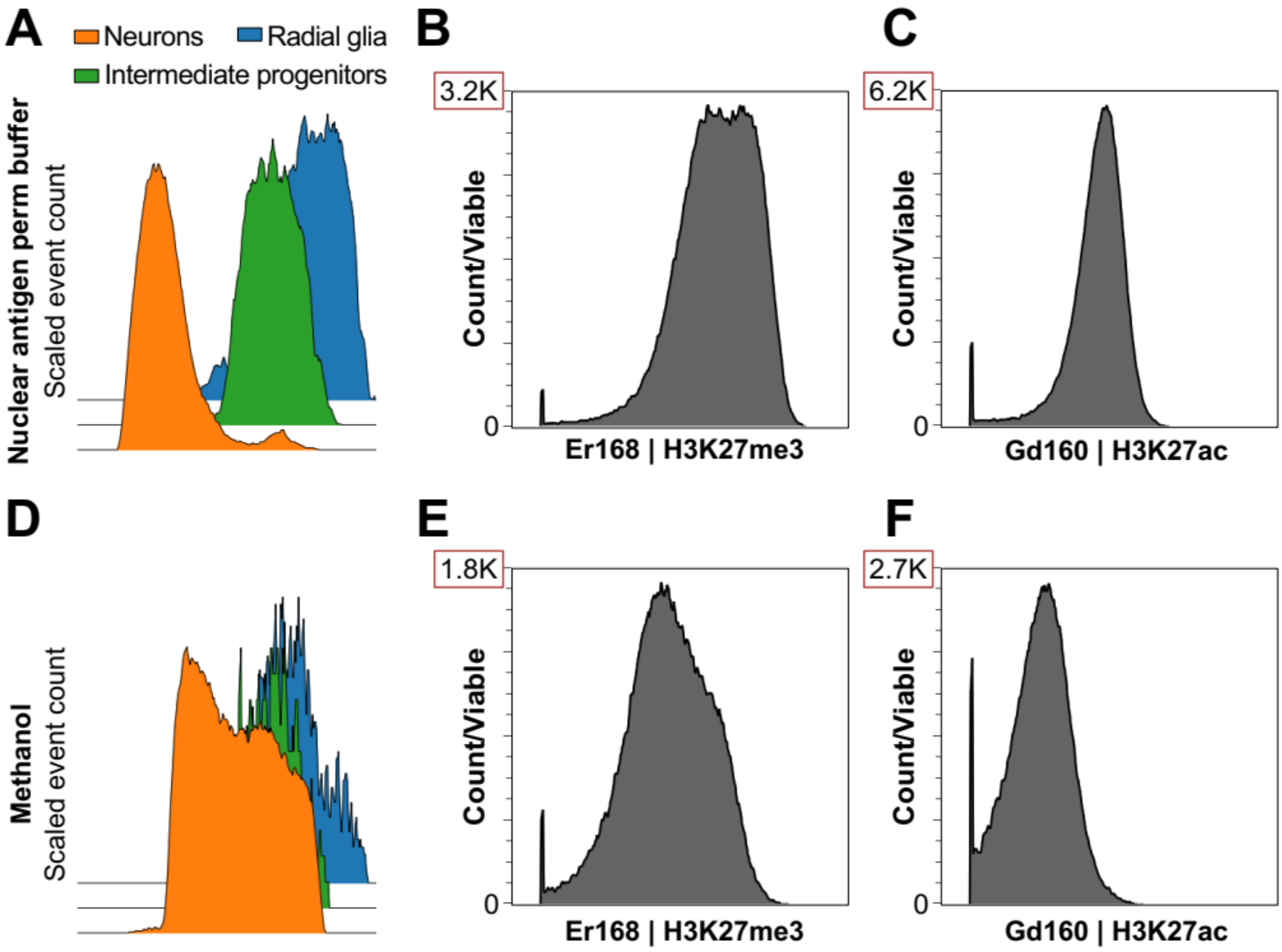

Supplementary Figure S3. Comparison of cell permeabilization with methanol versus a commercially available Nuclear Antigen Permeabilization Buffer. A) Histogram overlay showing cell populations that are manually gated according to lineage-specific cell type markers with Nuclear Antigen Perm Buffer permeabilization. **B-C)** Histograms show the expression of H3K27me3-^{168}Er **(B)** and H3K27ac-^{160}Gd **(C)** after Nuclear Antigen Perm Buffer permeabilization. **D)** Histogram overlay showing cell populations that are manually gated according to lineage-specific cell type markers with methanol permeabilization. Overall distribution of cell populations is disrupted. **E-F)** Histograms show the expression of H3K27me3-^{168}Er and H3K27ac-^{160}Gd after methanol permeabilization. The red boxes in **B**, **C**, **E**, and **F** indicate the number of viable cells (**related to Step 18)**.

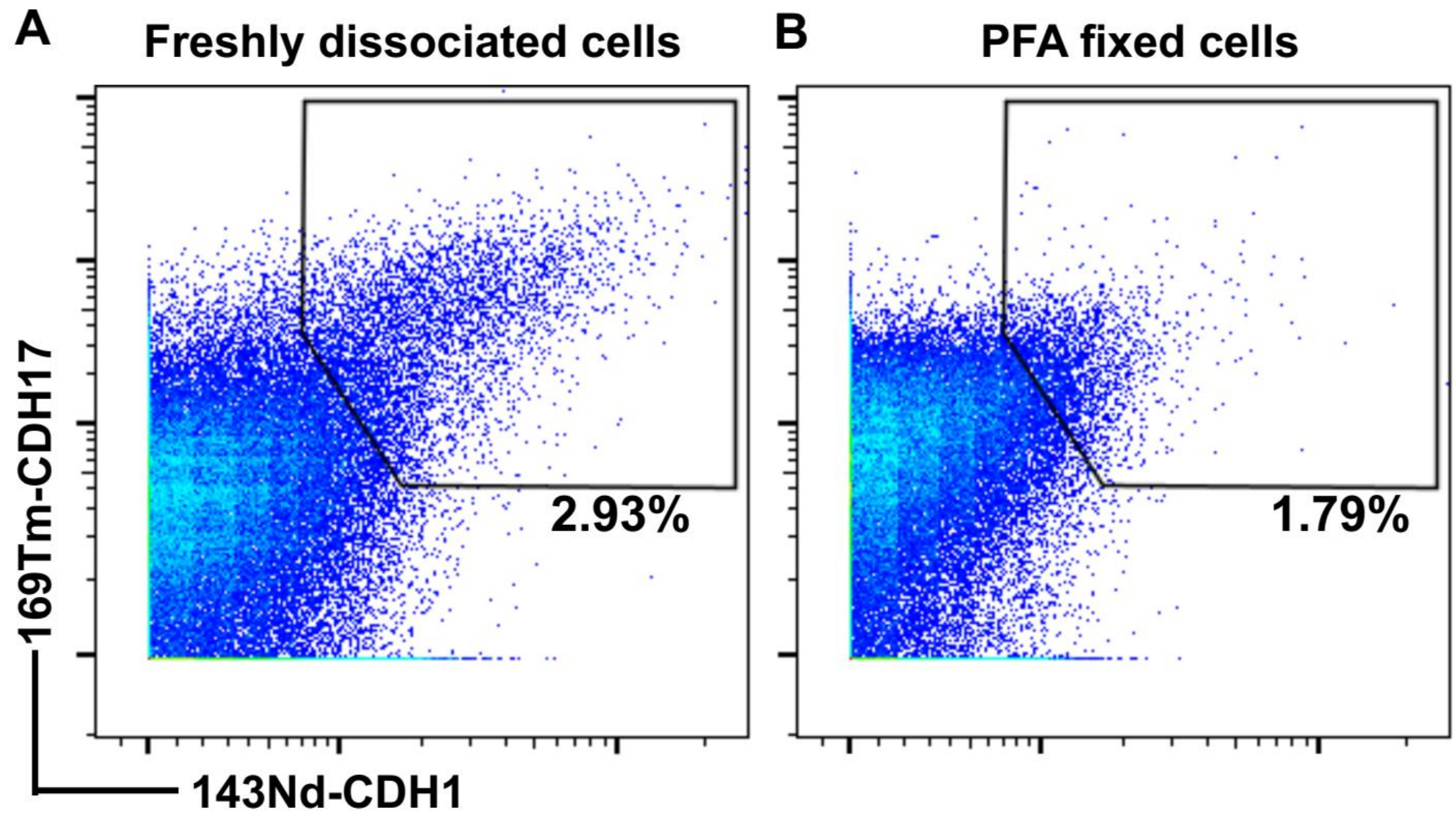

Supplementary Figure S4. Extracellular staining of gastro-esophageal tumoroid cells with the epithelial lineage markers CDH1 (E-cadherin) and CDH17 (cadherin 17), freshly after dissociation and after PFA fixation. A) In freshly dissociated gastro-esophageal tumoroids, the frequency of double-positive $CDH1^{+}CDH17^{+}$ cells is 2.93%, whereas **B)** this drops to 1.79% after PFA fixation. This indicates that PFA affects the CDH1 and CDH17 epitope structure and reduces the antigen detection with the used antibody clones. Therefore, the experimental samples are all stained fresh for CDH1 and CDH17 before PFA fixation (**related to Step 21**).

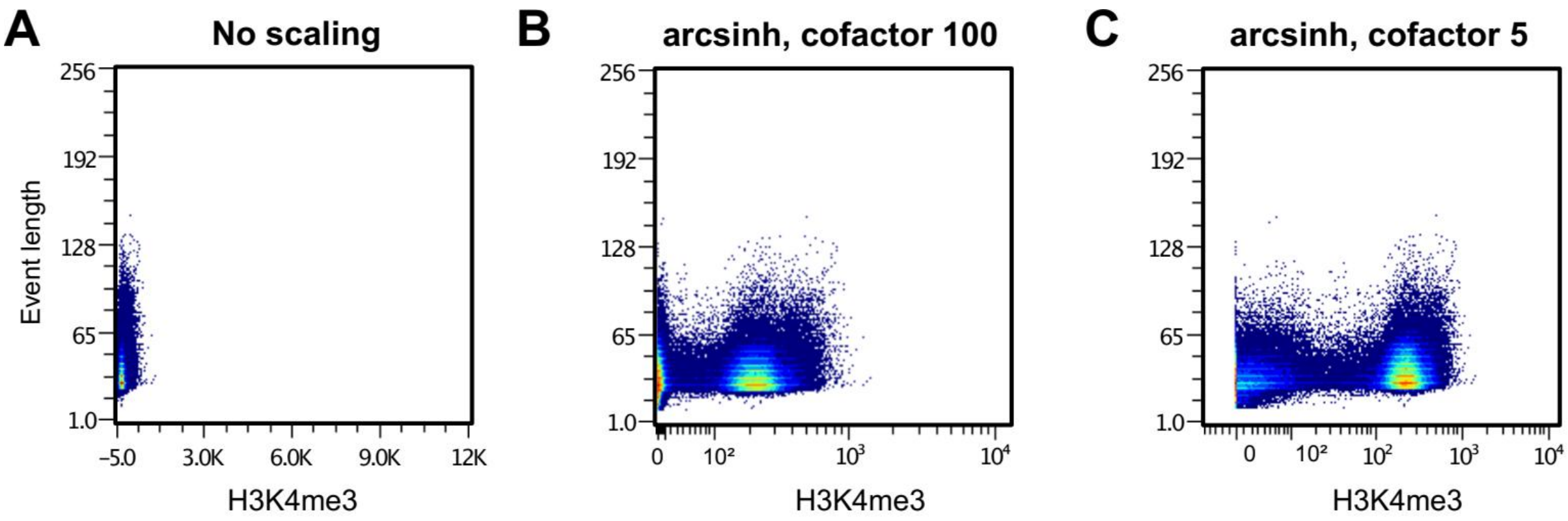

Supplementary Figure S5. Influence of data scaling after hyperbolic arcsine (arcsinh) transformation on data display. A) H3K4me2/3 signal without prior scaling. **B)** H3K4me2/3 signal transformed with arcsinh with a cofactor of 100. **C)** H3K4me2/3 signal transformed with arcsinh with a cofactor of 5. The arcsinh transformation is linear at lower values, corresponding to events close to zero, and logarithmic at higher intensities. The scaling factor, often called the cofactor, defines the width of this linear region around zero. For CyTOF data, a cofactor of 5 is recommended (**related to Step 31)**.

Supplementary Table

Supplementary Table S1. Ingredients and final concentrations of all components used to prepare the gastro-esophageal organoid culture medium, including the basal medium, growth factors, signaling pathway modulators, supplements, and other additives.

REAGENT	FINAL CONCENTRATION	AMOUNT
A83-01	0.5 µM	10 µL
B27	1X	1 mL
DMEM/F12	n.a.	11.590 µL
FGF10	200 ng/mL	100 µL
GlutaMAX	1X	500 µL
HEPES	10 mM	500 µL
[Leu^{15}] Gastrin	1 nM	50 µL
Mouse recombinant EGF	50 ng/mL	50 µL
N-acetyl-L-cysteine	1.25 mM	100 µL
Nicotinamide	10 mM	500 µL
Noggin-conditioned medium	10%	5 mL
Primocin	100 µg/mL	100 µL
Penicillin-streptomycin	100 U/mL (20 µg/mL)	500 µL
R-spondin1 conditioned medium	10%	5 mL
Wnt3A conditioned medium	50 %	25 mL
**Total**		**50 mL**
